# Will It Survive? Evaluating the Effects of Damage and Silviculture on Tree Seedling Survival Using Multi‐State Models

**DOI:** 10.1002/ece3.73496

**Published:** 2026-05-06

**Authors:** Emilie Champagne, Daniel Dumais, Geneviève Picher, Patricia Raymond

**Affiliations:** ^1^ Direction de la recherche forestière Ministère des Ressources naturelles et des Forêts Ville de Québec Canada

**Keywords:** adaptation, assisted migration, forestry, illness‐death models, plantation, silviculture

## Abstract

Plantations are the spearhead of adaptive silviculture, as planting stock can be selected to increase the resistance and resilience to climate‐related stressors and to accelerate the transition toward future conditions. Yet, plantations require high investments for an uncertain future outcome. Especially, we do not have a global understanding of how damage from abiotic and biotic agents relates to seedlings’ survival, and how ‘damage history’ can be modulated by silvicultural treatments. Here, we aimed at establishing the effects of timing and frequency of damage (i.e., loss of photosynthetic tissue, mechanical damage, herbivore damage, or presence of pathogens) on the survival of planted trees, using 6 years of surveys in an assisted migration experiment. Seedlings of eight species were categorized as ‘Healthy’, ‘Damaged’, or ‘Dead’ each fall. We analyzed transitions among categories using multi‐state models, a technique typical of the medical field that can be used to study disease progression. Survival and recovery of damaged seedlings declined with earlier and more frequent damage for all species, with the exception of *Pinus strobus* , affected by white pine blister rust. Shade‐intolerant species suffered less damage and had reduced mortality and higher recovery rates in the patch clearcut (1.2 ha), when compared to the shelterwood cut (40% of basal area removed). Shade‐tolerant species benefitted from open light conditions, either because these conditions were appropriate for a quick establishment or because of nursery's full light conditions. These results highlight the importance of providing adequate environmental conditions for quick seedling establishment, which will affect seedlings' ability to withstand damage. Although multi‐state models have limits, we propose that the study of ‘damage history’ can provide valuable insights in the context of plantations and could be expanded to other uses. Damage and recovery rates, especially, could be used as early indicators of plantation success, guiding the choices of silvicultural treatments.

## Graphical abstract

Plantations are the spearhead of adaptive silviculture, yet we do not understand how damage from abiotic and biotic agents relates to seedlings’ survival. In this study, we used multi‐state models, which originate from the medical field, to evaluate the relation between ‘damage history’, silvicultural treatments, and planted tree survival. We propose that the study of damage history can bestow valuable insights by providing early indicators of plantation success and could be expanded to other uses.

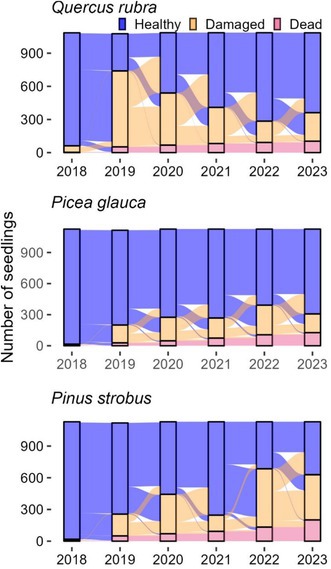

## 1 Introduction

“*There is no management practice in forestry that requires such a commitment to the future as plantation establishment*.”‐Margolis and Brand (1990)

In the temperate and boreal forests of the northern hemisphere, the tree plantations established when this sentence was published, some 35 years ago, are still in their infancy and have not produced a financial benefit. Yet, considerable energy and resources were required to collect seeds, to produce seedlings, to plant, and to maintain plantations. Even though plantations take a long time to generate forests and provide ecosystem services such as wood, they are the spearhead of adaptive silviculture, a tool aimed at maintaining productive forest ecosystems in the context of global change (Nagel et al. 2017; Thiffault et al. 2021; Clark et al. 2023). Namely, plantations can be designed to augment the resistance and resilience of managed forests by selecting a diverse planting stock able to tolerate climate‐related stressors (Messier et al. 2021). Moreover, by translocating populations and species, we can use plantations to accelerate the transition toward future conditions, a method called forest‐assisted migration (Millar et al. 2007; Pedlar et al. 2012). Plantations are also a cornerstone of mitigation, with the possibility to select planting stock for their carbon capture abilities (Clark et al. 2023). To make appropriate choices for the future, we need to develop early risk indicators that will compensate for the lack of information generated by trees' slow growth.

Plantation success is generally defined by survival and growth rate, which are linked to the ability of tree seedlings to overcome plantation stress and get established (Grossnickle 2005). Notwithstanding the effects of planting stock quality, plantation stress can be minimized by selecting sites or preparation methods providing an appropriate regeneration niche that will provide the species‐specific requirements in water, light, and soil conditions (Grossnickle 2000; Luoranen et al. 2025). The use of appropriate silvicultural treatments contributes to the creation of these conditions (Margolis and Brand 1990); for example, shade‐intolerant species will benefit from harvests that remove large proportions of the overstory (Cogliastro and Paquette 2012). Establishment also relies on site conditions, but especially conditions promoting root growth, which will provide proper access to soil water and nitrogen (Margolis and Brand 1990; Grossnickle 2000). Moreover, even if nursery‐grown seedlings have good reserves, root growth is correlated to photosynthesis (Burdett 1990). Thus, any event that damages the root system or the photosynthetic biomass of newly planted seedlings could enhance plantation stress, delay establishment, and consequently reduce plantation success.

Numerous abiotic and biotic agents can damage planted seedlings, such as late or early frost, herbivores, and diseases. Most studies regarding damage to planted seedlings are interested in life‐threatening events and thus investigate the immediate impacts on survival. For example, the effect of a drought event on the survival and growth of planted seedlings has frequently been evaluated (e.g., Tear et al. 1982; Guo et al. 2010; Chang et al. 2020), probably because of the critical effect of water availability before establishment. Other damage types, such as loss of photosynthetic and/or structural tissue by herbivores, have also generated interest (e.g., O'Reilly‐Wapstra et al. 2014; Ameztegui and Coll 2015; Redick et al. 2020). In recent years, late frost and winter desiccation, which can damage photosynthetic tissue or buds, have gathered a lot of interest, as they could be particularly critical when translocating provenances and species (e.g., Benomar et al. 2022; Mura et al. 2022; Luoranen et al. 2025). Little is known about the combination of several damage types and their timing on planted trees (but see Luoranen et al. 2025). We could gather a better understanding of cumulative risk factors over time by getting inspiration from the medical field, where researchers have investigated thoroughly the effect of diseases or accidents on life expectancy using longitudinal datasets, for example, repeated health assessments on a group of individuals.

**FIGURE 1:**
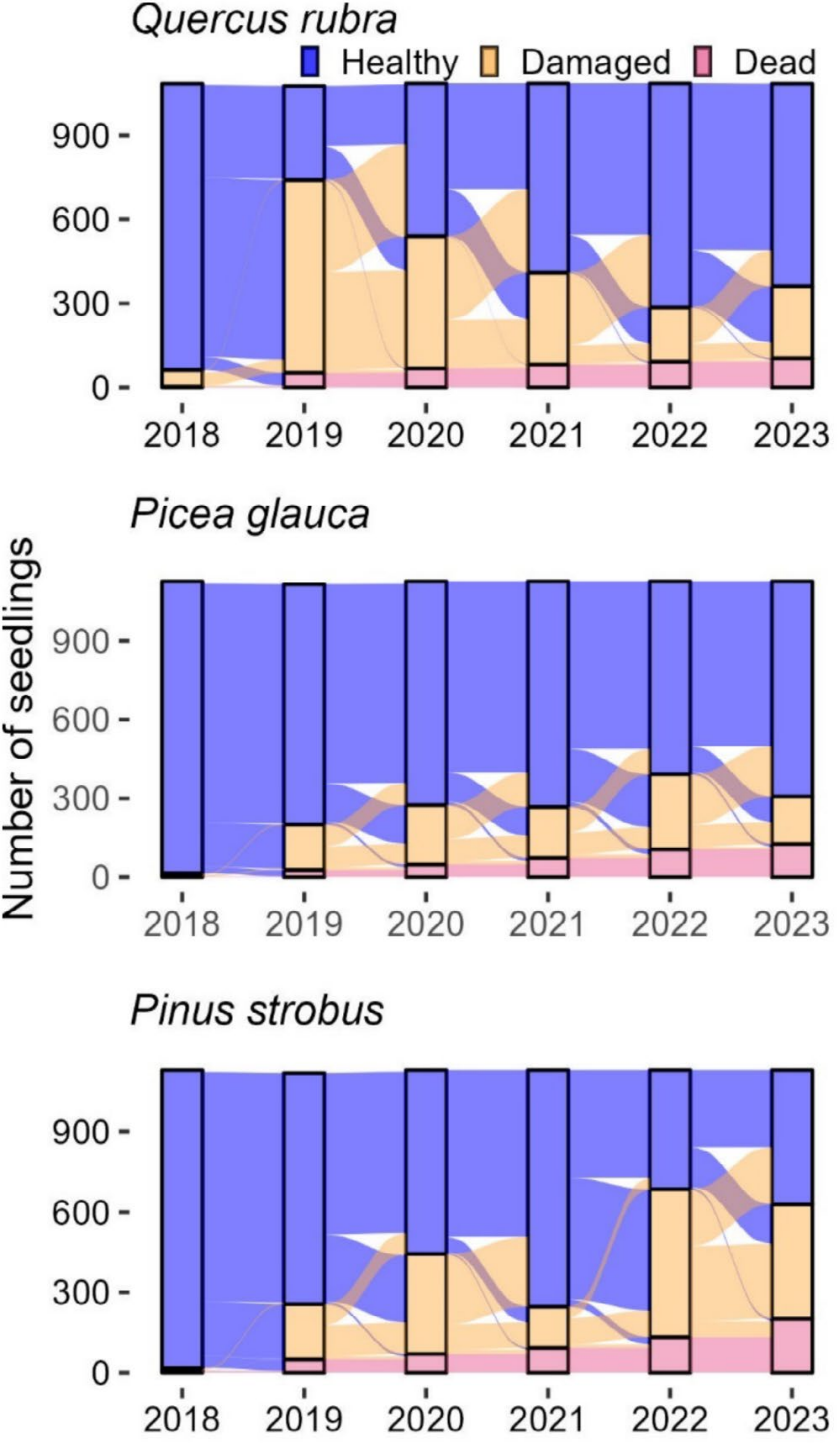
Sankey style diagram presenting the ‘damage history’ of three species planted in the Lac‐des‐Amanites experiment, from 2018 to 2023. Each fall, seedlings were visually classified either as ‘Healthy’, ‘Damaged’, or ‘Dead’. ‘Damaged’ referred to multiple possible issues with the potential to affect photosynthetic capacity or survival (see Table 1). Columns above years indicate the number of seedlings in each category at the fall survey, and lines among columns indicate transitions among states (e.g., a blue line between 2018 and 2019 ending in the orange section indicates ‘Healthy’ seedlings that transitioned to a ‘Damaged’ state). *Quercus rubra* is representative of the deciduous species: Lots of damage early, decreasing with time since plantation. *Picea glauca* exemplifies the conifers' damage history: Relatively stable proportion of damaged seedlings. *Pinus strobus* , a species susceptible to white pine blister rust, presents a different history, with an increase in damage with time. There are multiple possibilities to analyze damage history patterns: Five‐year survival, regression models, and so on. We opt to focus on the transitions between states and how silvicultural treatments and damage history influence them.

Here, we mainly aimed to establish the effect of damage (i.e., loss of photosynthetic tissue, mechanical damage, herbivore damage, or the presence of pathogens) on the survival of planted trees. More specifically, we asked: (1) What is the effect of damage frequency and timing on survival? and (2) Can silvicultural treatments modulate the impact of damage on survival? We hypothesized that earlier and recurring damage (the same or another type) would have a stronger negative impact on survival, as they occur during early establishment. We also hypothesized that modifications of the environment via silvicultural treatments could improve the ability of seedlings to recover, by providing adequate resources for establishment. For example, damaged shade‐intolerant seedlings should present a lower mortality rate and a higher recovery rate in open conditions in comparison with partially sub‐optimal shaded conditions. We investigated these questions using a 6‐year survey of an assisted migration plantation, and with statistical analyses that have been developed in the medical field to study disease progression (Jackson 2011). We previously found a very high overall survival rate after 5 years (84%; Raymond et al. 2025), but we also observed multiple types of damage and patterns in their occurrence that have not yet impacted survival (Figure 1). We thus hoped that a temporal analysis of damage could provide early indicators of interventions reducing damage risk or improving recovery, with the objective of maximizing plantation success.

## 2 Materials and Methods

### 2.1 Site and Study Design

We used data from the Lac‐des‐Amanites site (Québec, Canada), an experimental part of the Desired Regeneration through Assisted Migration (DREAM) network (Royo et al. 2023). DREAM is also a framework aimed at reducing uncertainties in forest‐assisted migration via empirical data. Projects in the network are experimental mixed‐species plantations of future‐adapted seed stock selected using climate modeling, with factorial manipulation of abiotic and biotic filters by operational silviculture treatments. A full description of the framework is available in Royo et al. (2023), with additional information regarding the Lac‐des‐Amanites site, including seedlots provenances, in Champagne, Royo, et al. (2021), Champagne, Turgeon, et al. (2021), Dumais et al. (2025) and Raymond et al. (2025).

**FIGURE 2:**
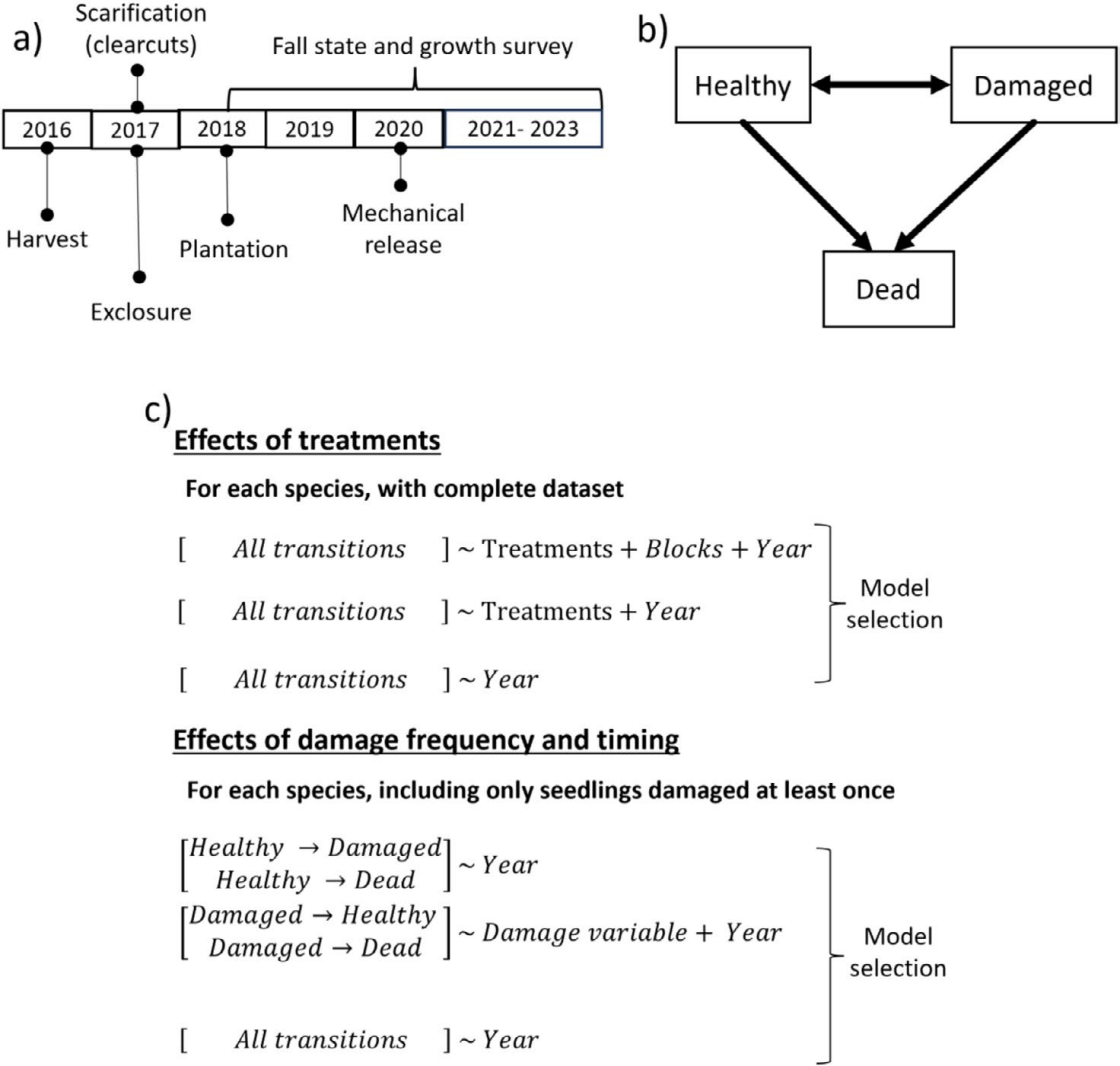
(a) Sequence of treatments and surveys at the Lac‐des‐Amanites experimental site; (b) Transitions among states allowed in the multi‐state model. In each survey, seedlings could either be ‘Healthy’ (no visible sign of damage), ‘Damaged’ (see Table 1), or ‘Dead’. Dead is a final, absorbing state from which no transitions are possible, whereas seedlings can transition between ‘Healthy’ and ‘Damaged’ states any number of times. Seedlings can also remain in a state for several surveys; (c) Statistical analysis process to evaluate the effect of treatments (top) and the effect of damage frequency and timing on transitions (bottom). In the models at the top, the covariate effects are evaluated for all transitions, whereas in the bottom models, covariates differ among transitions. The damage variable refers to two variables and hence two models.

The Lac‐des‐Amanites site is in the Réserve faunique de Portneuf (Lat. 47.128° N, Long. −72.409° W), at the southern edge of the balsam fir ( *Abies balsamea* )‐yellow birch ( *Betula alleghaniensis* ) bioclimatic domain (Saucier et al. 2009). This managed forest is located within a wildlife reserve, where silvicultural systems using partial cutting predominate, but prior to the 1990s, diameter‐limit cutting was widespread (Royo et al. 2023). Briefly, the experiment is a full split‐split‐split factorial design including two levels of an overstory cutting treatment (a uniform shelterwood cut with 40% of merchantable basal area removed and a 1.2 ha patch clearcut), a cervid exclusion treatment (fenced and unfenced), and a competing vegetation treatment (present and removed once via mechanical release with brushsaws). Fences were made of woven galvanized wire (2.4 m tall), excluding cervids, here white‐tailed deer ( *Odocoileus virginianus* Zimmermann) and moose ( *Alces alces* L.). This design is replicated in four blocks. Treatment application sequence is summarized in Figure 2a (see Royo et al. 2023 for a schematic of the experimental design). A climate analogue treatment with three levels is nested within the factorial combination of the other treatments (cutting × cervid exclusion × competing vegetation × analogue = 24 experimental units per block). Climate analogues are different seed provenances, each a location where the current climate is analogous to the future climate predicted at the plantation site. Here, one analogue is of the current climate, with the other two representing climate predicted for mid‐century (1941–1970) and end‐of‐century (1971–2100) at the present experimental site (see Raymond et al. 2025 for climate analogue modeling parameters). In the experimental units, seedlings are planted in 12 rows, each including nine species, placed in a random order.

Species were selected to represent a diverse mix of coniferous and deciduous species, with ecological and economic values, but also with varying habitat suitability predicted for the end of the century. Because of predicted changes to the climate, three are expected to have a decreasing habitat suitability ( *Picea glauca* , *Picea rubens* , and *Thuja occidentalis* ), whereas three others are expected to have an increasing habitat suitability ( *Pinus strobus* , *Pinus resinosa* , and *Acer saccharum* ). The latest three are newcomers in the experimental area that could experience a range expansion into this bioclimatic domain ( *Prunus serotina* , *Quercus rubra* , and *Carya ovata* ). Here, we will not be discussing *Carya ovata* , as it was planted in a different year because of the longer production time required. For the rest of this article, species will be presented in the rough order of their general shade tolerance, from the most to the least tolerant: *Picea rubens* , *Acer saccharum, Thuja occidentalis, Picea glauca*, *Quercus rubra* , *Pinus strobus* , *Prunus serotina* , and *Pinus resinosa* (Ministère des Ressources naturelles 2013). All seedlings were produced at the same facility, a provincial government nursery (Berthier, QC, Canada) with coniferous seed sown in 2017 and deciduous in 2018 in containers (large stock seedlings with 310 cm^3^ and 340 cm^3^ cell volume, respectively). We planted all seedlings in August and September 2018, each seedling separated by 2 m; 2 m among seedlings in a row and among rows for a maximum of 108 seedlings per experimental unit. A maximum of 1152 seedlings per species was thus planted, although actual numbers are slightly lower because of the vagaries of the production and plantation process (Table 1).

**TABLE 1:** Damaged type description and frequency, that is, number of times the damage was recorded by species. Frequencies are summed over the entire survey period (5 years), with the possibility to have more than one damage type per year.

Damage type	Description	*Picea rubens*	*Acer saccharum*	*Thuja occidentalis*	*Picea glauca*	*Quercus rubra*	*Pinus strobus*	*Prunus serotina*	*Pinus resinosa*
	N of seedlings	1122	1074	1135	1123	1082	1123	1087	1123
	N of individuals damaged at least once (% of all individuals)	744 (66%)	879 (82%)	693 (61%)	535 (48%)	937 (87%)	874 (78%)	807 (74%)	578 (51%)
Animal damage	Damage by animals not covered by another category (e.g., debarking)	0	8	1	0	3	3	7	0
Browsed	Browsing by white‐tailed deer, moose or snowshoe hare, either on the leader or lateral twigs	428	345	412	225	296	675	339	474
Broken	Broken leader	19	257	25	6	176	68	242	27
Burnt	Frost or heat burns	34	379	238	19	70	22	74	11
Crushed or bent	Seedling has been crushed or bent. Cause can be known (e.g., windthrow of a tree on a seedling) or unknown and suspected (snow)	76	78	112	44	80	114	112	146
Dead leader	Leader is dead, for reasons not identified/covered by another category	315	993	226	131	1332	152	736	91
Insects and diseases	Signs of insects or diseases	108	154	3	231	173	789	158	16
Needle or leaf loss	Loss of leaves or needles, for reasons not identified or because of defoliators	702	50	363	507	127	144	140	133
No budburst	Buds never opened	80	24	6	64	31	14	7	20
Uprooted	Root visible, usually because of frost/defrost cycles (frost heaving)	6	2	2	3	2	1	2	0
Wound	Broken branches not identified/covered by another category	8	33	11	12	56	69	46	62

### 2.2 Seedling State Survey

Starting in the plantation year (Figure 2a), we performed an annual fall survey (*n* = 6) of all planted seedlings, where the height, diameter 1 cm above ground‐level and state were recorded (See Raymond et al. 2025 for growth and survival analyses). Seedlings state was defined either as ‘Healthy’, ‘Damaged’, or ‘Dead’. The ‘Damaged’ state referred to multiple possible issues, but for the sake of this study we only considered damages with the potential to affect photosynthetic capacity or survival (Table 1), that is, seedlings had either loss photosynthetic biomass, presented trace of an active/recent pest or pathogen, had significant damage to the main stem or were affected by abiotic factors. Seedlings that could not be found, usually because of the growth of competing vegetation, were considered dead. For the purpose of this study, we removed from the dataset seedlings that disappeared or were recorded dead and reappeared as healthy or damaged (*n* = 101), because of the uncertainty regarding their damage history. We kept, however, seedlings for which one or more survey state value was not evaluated (81 missing states), usually indicating a lack of access (wasp nest) or an oversight during the survey; an experimental unit was forgotten and not surveyed in 2019. Finally, we removed seedlings that were sampled for an ecophysiological study (Dumais et al. 2025) or accidentally damaged by human action (*n* = 193), including damage that occurred during the mechanical removal of competing vegetation.

### 2.3 Statistical Analyses

To answer our questions about the effect of treatments on damage, and on the relation between damage and survival, we used multi‐state models. Multi‐state models are used to model transition rates of study subjects among several states in longitudinal datasets (Gentleman et al. 1994; Jackson 2024). They are typically used in the medical field to study disease progression, where states are the different stages of a disease, including an absorbing state (death) that once reached is final. Multi‐state models are extensions of survival analyses where there are only two states (alive, dead) with unidirectional transitions (Eulenburg et al. 2015; Groha et al. 2021). In a multi‐state model, we can specify which transitions between states are possible (including bi‐directional transitions) and transitions are modeled with transition intensity functions (Jackson 2024). Here, we used a transition structure also called illness‐death model (Eulenburg et al. 2015), where seedlings can transition between ‘healthy’ and ‘damaged’ states, or to an absorbing ‘dead’ state (Figure 2b). All models were created using the ‘msm’ function of the msm package (version 1.8; Jackson 2011) in R 4.4.1 (R Core Team 2024). This function allows the specification of categorical and continuous covariates (i.e., explanatory variables) that can influence transition intensity functions; these covariates can be constant or vary with time (Jackson 2024). Covariates can be applied to all transition intensity functions or to a subset (e.g., only for ‘Damaged → Dead’ transitions; Jackson 2024).

#### 2.3.1 Effects of Treatments on Transitions

We created multi‐state models with treatments as covariates (*Cutting*, *Cervid exclusion*, *Competing vegetation, Analogues*; hereafter treatments models). Each species was analyzed separately. Transitions toward death were relatively infrequent (Table 2), which is problematic for model convergence and calculations of estimates (Eulenburg et al. 2015; Jackson 2024), especially if a small number of transitions are divided among multiple categorical variables. To reduce convergence issues and unreliable estimates from a more complex model structure, we did not include interactions among treatments and used a model selection approach for simplification (see Figure 2c for analysis process). We compared the AIC of treatments models with and without *Blocks* and selected the model with the lowest AIC (Burnham and Anderson 2002). We considered models with delta AIC < 10 to be equivalent and report the results of the model with the lowest number of covariates (Burnham and Anderson 2002; Mazerolle 2006). We also compared models using the likelihood ratio test (Eulenburg et al. 2015). Additionally, the msm function cannot accommodate random effects, thus our only option to account for the effects of blocks was to include it as a covariate (*Blocks*); consequently, the models' result has a limited inference ability and is specific to these four blocks. If *Blocks* was present in the selected model, we do not report its effect on transitions because we were not interested in block differences per se.

**TABLE 2:** Number of transitions (% of all transitions per species in parentheses) among states (‘Healthy’, ‘Damaged’, or ‘Dead’), all years combined, per species.

		To state		
	From state	Healthy	Damaged	Dead
*Picea rubens*	Healthy	2888 (57%)	861 (17%)	126 (2%)
	Damaged	678 (13%)	492 (10%)	56 (1%)
*Acer saccharum*	Healthy	2089 (42%)	1062 (21%)	93 (2%)
	Damaged	913 (18%)	732 (15%)	77 (2%)
*Thuja occidentalis*	Healthy	3292 (63%)	817 (16%)	82 (2%)
	Damaged	581 (11%)	375 (7%)	64 (1%)
*Picea glauca*	Healthy	3669 (68%)	722 (13%)	79 (1%)
	Damaged	509 (9%)	337 (6%)	44 (1%)
*Quercus rubra*	Healthy	2060 (40%)	1259 (25%)	64 (1%)
	Damaged	1026 (20%)	680 (13%)	35 (1%)
*Pinus strobus*	Healthy	2777 (53%)	1117 (21%)	97 (2%)
	Damaged	603 (11%)	595 (11%)	97 (2%)
*Prunus serotina*	Healthy	2036 (44%)	1124 (24%)	178 (4%)
	Damaged	813 (17%)	458 (10%)	45 (1%)
*Pinus resinosa*	Healthy	3587 (71%)	636 (13%)	121 (2%)
	Damaged	417 (8%)	221 (4%)	70 (1%)

In each of the treatments models (with and without *Blocks*), we included year as a covariate for all transitions (Jackson 2025), because seedling establishment is a time‐dependent process, that is, we can expect seedlings to die less frequently as they age and become established. This is a crucial covariate to include, as multi‐state models assume a time‐homogenous process, that is, transition between two states at time *t* + 1 depends only on the state the subject is in at time *t* (Gentleman et al. 1994; Jackson 2024). Again, to avoid needless complexity, we compared treatments models (with or without *Blocks*) to a null model including only year, using AIC values and the log‐likelihood ratio test (Burnham and Anderson 2002; Eulenburg et al. 2015). For the resulting three candidate models, we added a scaling factor to normalize likelihood, using the log‐likelihood value of the more complex model, as described in Jackson (2024; ‘fnscale’ argument). As the state survey are done once a year in fall, we did not have exact transition time among states, a situation known as ‘panel‐observed’ data; we thus parametrize the msm function for panel data (obstype = 1).

We tested model sensitivity to the choice of initial value for the transition intensity matrix (also named Q matrix), which defines the possible transitions among states in continuous time. We defined the initial Q matrix using values of 1 for all allowed transitions, as proposed by Jackson (2025). We used the most complex model (Treatments *+ Blocks + Year*) to compare results with models using Q matrix values of 0.01 and 3, respectively. We also evaluated model fit by comparing visually the predicted and observed values (Gentleman et al. 1994, Jackson 2024). Because, again, of the low occurrence of some transitions and high complexity of our models, the evaluations of model fit provided by formal tests were unreliable (Jackson 2024).

#### 2.3.2 Effects of Damage Frequency and Timing on Transitions

To evaluate the effect of damage frequency and timing on transitions, we kept only seedlings that were damaged at least once. Species were also analyzed separately. We tested the effects of two covariates on the transitions from ‘Damaged’ to ‘Healthy’ (hereafter ‘Damaged → Healthy’) and from ‘Damaged to ‘Dead’ (hereafter ‘Damaged → Dead’; Figure 2c). The first damage covariate added was the *Number of times in state ‘Damaged’*, a variable that was continuous and progressive (i.e., adjusted at each transition on the basis of previous damage). The second variable is the *First year in state ‘Damaged*’, which was also continuous but constant. These two variables could not be included in a single model, as they were collinear (R^2^ > 0.30), generating two models (hereafter called damage models). We could not use a model selection approach to compare these models to the treatment models, because they used a subset of the entire dataset (Mazerolle 2006). We also compared, however, the damage models to a year‐only null model. As for the evaluation of the effect of treatments, *Year* was also included as a covariate to account for the time‐inhomogeneous process in the damage models. The models also evaluated the transition intensity function for the two other transitions (‘Healthy → Damaged’ and ‘Healthy → Dead’) but only with the *Year* covariates.

We performed the same adjustments as described in the previous section. When choosing a factor to normalize the likelihood, we used a log‐likelihood value between the two damage models. We assessed model sensitivity to the initial Q matrix values using the model with the *Number of times in state ‘Damaged’* for comparison.

#### 2.3.3 Interpretation of the Results

To assess the effects of treatments and damage on transitions, we used the hazard.msm function, that produce hazard ratios (HR) with 95% confidence intervals (CI). An HR < 1 indicates a lower transition intensity (reduced instantaneous risk), whereas an HR > 1 indicates a higher transition intensity, all relative to a reference level when considering categorical variables. For models evaluating the effects of treatments on transition, we set the reference levels to clearcut, fenced, competing vegetation removed, and current climate analogues. We considered CI that did not include 1.0 to be statistically significant; we rounded values on the basis of the significant numbers in the CI. We present HR back transformed from the log scale in tables, which are directly interpretable as changes in hazard (i.e., a HR of 1.2 indicates a 20% increase in instantaneous risk). In figures, we give HR on the log‐scale, as back‐transformed CI are asymmetric and skew the axis. We present results from the best treatment model (lowest AIC, either Treatments *+ Blocks + Year* or Treatments *+ Year*) and from the two damage models. Although we compared the two damage models using AIC, we present the results of both, because each answers a different question (cumulative damage vs. timing of first damage). We give the estimates for the effect of *Year* from the treatment effect model, as this model included all seedlings, not only those who experienced damage.

## 3 Results

### 3.1 Model Comparison and Fit

On the basis of AICs and log‐likelihood ratio tests, models with treatments or damage variables were all more plausible than year‐only models (Tables S1 and S2). For all models tested, the results were identical for the three Q matrices' initial value. However, some estimates of the confidence interval (CI) were extremely large, especially for *Prunus serotina* and for the effects of the overstory cutting treatment (Table S3). Large estimates suggest an overcomplex model or data sparsity, i.e., rare transition types (Eulenburg et al. 2015). Mortality (‘Healthy → Dead’ and ‘Damaged → Dead’) is indeed infrequent in our dataset (Table 2); for example, these transitions represent only 5% of all transitions for *Prunus serotina* . The proportion of mortality can be further reduced when separating by treatment levels. For *Prunus serotina* , mortality was well divided between the shelterwood cut and the clearcut (113 and 110 transitions, respectively), but of those, most were ‘Healthy → Dead’ transitions (86 and 92 transitions, respectively). The ‘Damaged → Dead’ transitions were far less abundant (27 in the shelterwood cut, 18 in the clearcut), which could explain the difficulty in estimating CI. We cannot exclude model overfitting as the cause of poor CI estimates, but the visual comparisons of expected and observed values suggest the models are appropriate (Figures S1–S8). The combination of AIC comparisons, log‐likelihood ratio tests, and visual assessments of fit suggests that the models presented are representative of the dataset, but that caution must be exerted when considering large CIs.

### 3.2 Effect of Year on Transitions

**FIGURE 3:**
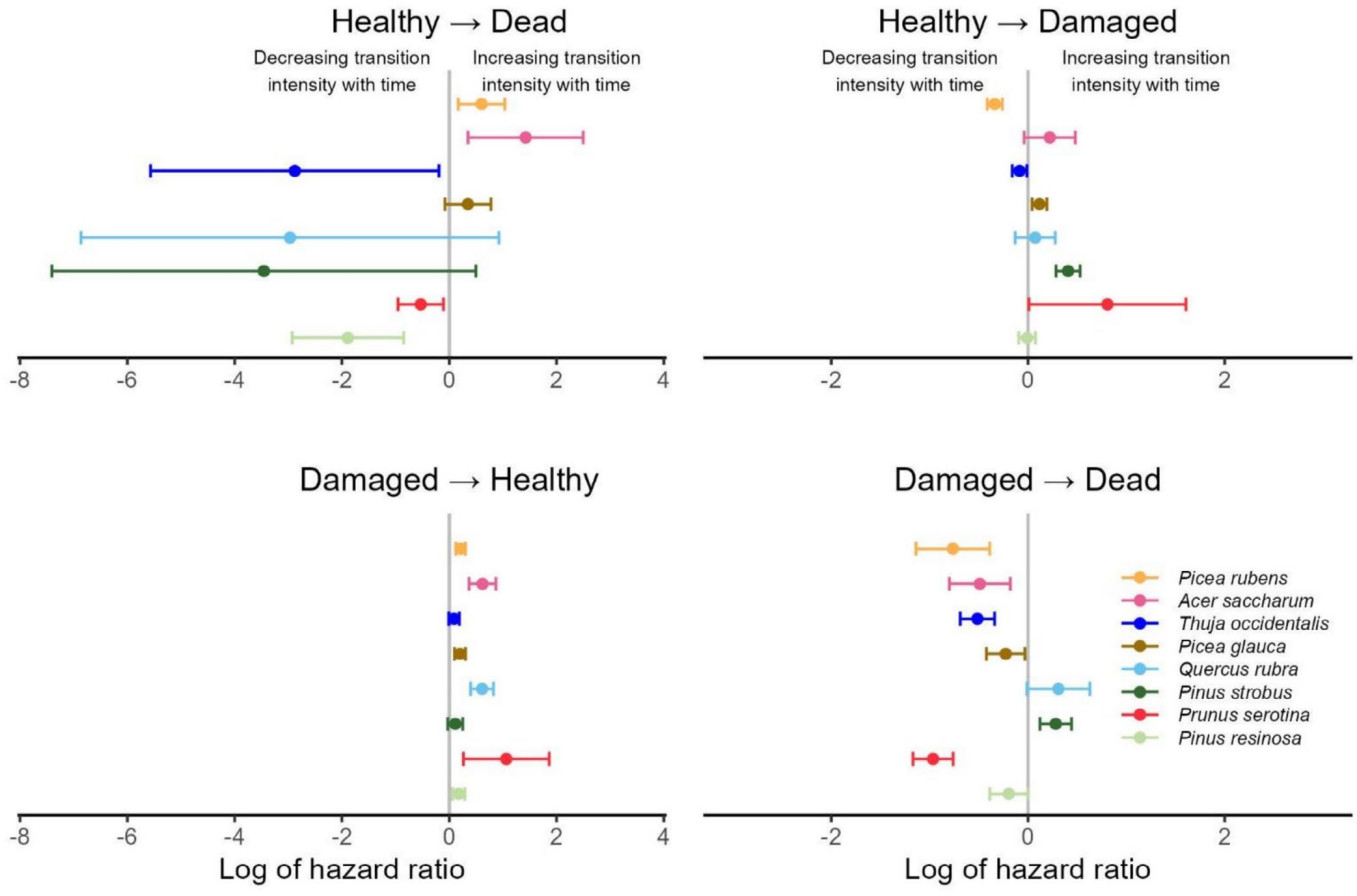
Log of the hazard ratio (HR) for the effect of the Year covariate, for each species, by transition type. Bars surrounding values are 95% CI. If the bar does not overlap 0, the HR is considered to be statistically significant. Species are ordered on the basis of their general shade tolerance, from the most to the least tolerant.

*Year* influenced the hazard ratio (HR) of at least one transition per species, and in most cases, of several transitions (Figure 3, Table S3), supporting our assumption that seedling establishment was a time‐dependent process. The general pattern is a decrease in mortality over time (‘Healthy → Dead’ and ‘Damaged → Dead’) and an increase in recovery (‘Damaged → Healthy’). More specifically, mortality of healthy seedlings decreased with time for *Prunus serotina* , *Pinus resinosa* , and *Thuja occidentalis* , although it did increase for *Acer saccharum* and *Picea rubens* . For *Picea rubens* , however, healthy seedlings were less damaged with time, recovered better, and damaged seedlings died less. Enhanced recovery with time was also present in five other species (Figure 3, Table S3); the HR was positive, but CI included 1.0 for the remaining two species ( *Pinus strobus* and *Thuja occidentalis* ). Moreover, the mortality of damaged seedlings decreased with time for four species (*Acer saccharum, Prunus serotina*, *Picea rubens* , and *Thuja occidentalis* ). For only one species, *Pinus strobus* , damage and mortality of damaged seedlings increased with time.

### 3.3 Effects of Treatments on Transitions

For most species, including *Blocks* in the model improved plausibility, on the basis of AIC values (Table S1); only for *Picea glauca* and *Pinus resinosa* , the model without the blocking effect was equally or more plausible. For *Quercus rubra* , the model with *Blocks* did not converge.

**FIGURE 4:**
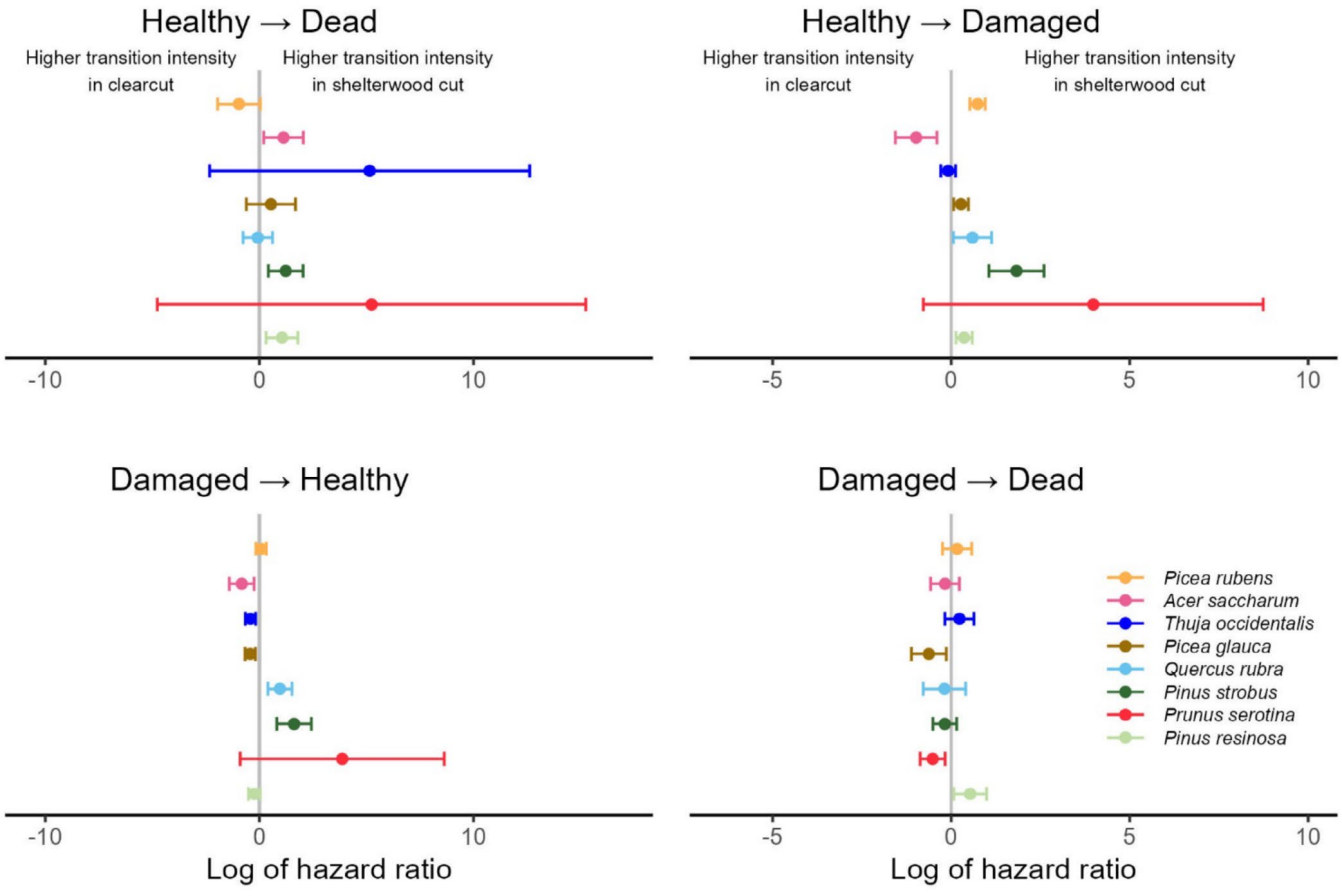
Log of the hazard ratio (HR) for the effect of the *Cutting treatment* covariate, for each species, by transition type. Bars surrounding values are 95% CI. If the bar does not overlap 0, the HR is considered to be statistically significant. Species are ordered on the basis of their general shade tolerance, from the most to the least tolerant.

The overstory cutting treatment seemed to have more influence on damage and mortality than other treatments, as all species had at least one statistically significant effect of this treatment on the HR of transition intensities (Table S3, Figure 4). The effects, however, are species dependent. Mortality of healthy seedlings was higher in the shelterwood cut than in the clearcut for three species ( *Acer saccharum* , *Pinus strobus* , and *Pinus resinosa* ), whereas cutting did not influence the HR of this transition for the five remaining species. Of these three species, two (the *Pinus*) also experienced higher damage rates in the shelterwood cut than in the clearcut. Although *Pinus strobus* benefitted from a higher recovery rate in the shelterwood cut, on the other hand, damaged *Pinus resinosa* had a higher mortality rate in this treatment (HR of 1.7 [1.1–2.7], a 70% increase in risk). Other species also presented higher damage rates for healthy seedlings in the shelterwood cut compared to the clearcut ( *Picea glauca* , *Picea rubens* , *Quercus rubra* ), but none presented a higher mortality rate for damaged seedlings in this treatment. On the contrary, damaged *Prunus serotina* and *Picea glauca* had a lower mortality risk in the shelterwood cut than in the clearcut. The effect of cutting treatment on recovery is more variable, with three species having higher recovery rates in the clearcut ( *Acer saccharum* , *Picea glauca* , and *Thuja occidentalis* ) and two having higher recovery rates in the shelterwood cut ( *Pinus strobus* and *Quercus rubra* ).

The effects of other treatments on damage and mortality rates are more scattered (Table S3). Large herbivore access led to a 70% higher damage rate for healthy *Thuja occidentalis* (HR = 1.7 [1.4, 2.1]) but to a 90% lower damage rate for healthy *Prunus serotina* (HR = 0.1 [0.0, 0.6]). Recovery (‘Damaged → Healthy’) of *Prunus serotina* and *Acer saccharum* was, respectively, 90% and 40% lower when large herbivores had access to the seedlings (HR of 0.1 [0.0, 0.5] and 0.6 [0.3, 0.9]). Competing vegetation presence had positive effects on two species. It reduced the damage rate of healthy *Thuja occidentalis* by 30% (HR = 0.7 [0.6, 0.9]) and increased the recovery rate of *Acer saccharum* by 170% (HR = 2.7 [1.1, 6.7]). However, competing vegetation presence had negative effects on *Picea glauca* , reducing its recovery rate by 30% (HR = 0.7 [0.6, 0.9]) and ambiguous effects on *Quercus rubra* and *Prunus serotina* . For *Quercus rubra* , competing vegetation presence increased damage rate by 160% (HR = 2.6 [1.5, 4.7]), although the recovery rate for this species was 170% higher in this treatment (HR = 2.7 [1.5, 5.0]). On the contrary, for *Prunus serotina* , competing vegetation reduced the damage rate by 80% (HR = 0.2 [0.0, 0.9]) but increased the mortality rate of damaged trees by 40% (HR = 1.4 [1.1, 2.0]).

Finally, we found very few differences among climate analogues: (1) *Quercus rubra* mid‐century analogue damage and recovery rates were, respectively, 150% (HR = 2.5 [1.2, 5.4]) and 160% (HR = 2.6 [1.2, 5.8]) higher than those of the current climate analogue; (2) *Prunus serotina* damaged seedlings' mortality rate was 60% (HR = 0.4 [0.3, 0.6]) lower for mid‐century analogue than for the current climate analogue; (3) the recovery rate of end‐of‐century *Acer saccharum* was 130% (HR = 2.3 [1.1, 4.9]) higher than those of the current climate analogue.

### 3.4 Effects of Damage Frequency and Timing on Transitions

**FIGURE 5:**
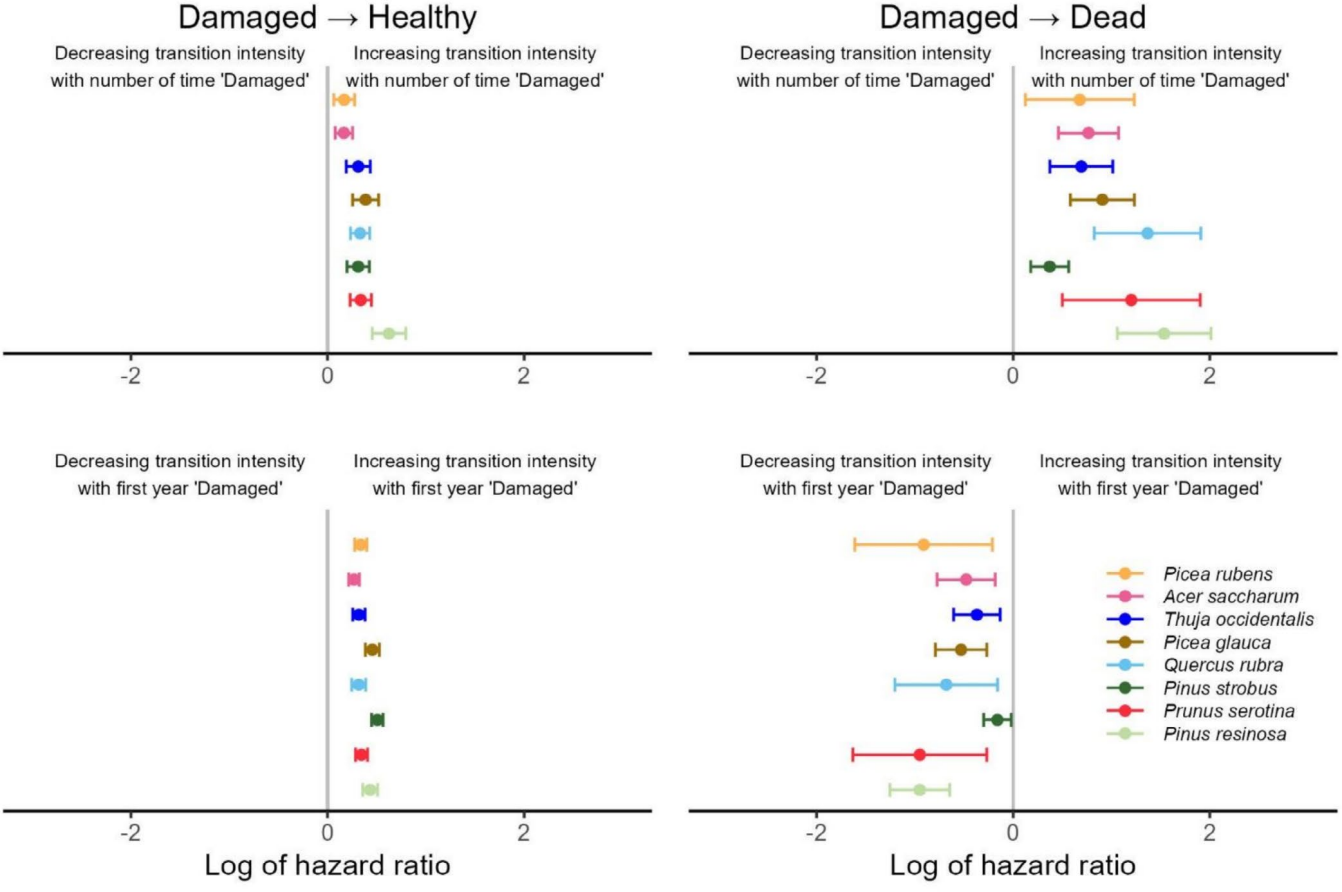
Log of the hazard ratio (HR) for the effect of the *Number of times in state ‘Damaged’* (top) and the *First year in state ‘Damaged’* (bottom) covariates, for each species, by transition type. Bars surrounding values are 95% CI. If the bar does not overlap 0, the HR is considered to be statistically significant. Species are ordered on the basis of their general shade tolerance, from the most to the least tolerant.

In contrast to the effects of treatments, the effect of damage frequency and timing was similar for all species (Table S4, Figure 5); higher damage frequency (*Number of times in state ‘Damaged’*) increased the recovery and mortality rates of damaged seedlings. The effect was stronger for mortality, ranging from 100% to 360% increases in HRs. When the first year of damage occurred later, it increased the recovery rate of all species. Late first damage also decreased the mortality rate, with the exception of *Pinus strobus* . Although we were interested in both frequency and timing, it is interesting to note that all AIC indicate that *First year in state ‘damaged’* provides a more plausible model than *Number of times in state ‘damaged’* (Table S2).

## 4 Discussion

Using an analytical framework typical of the medical field, we investigated the effects of silviculture and damage (e.g., breakage, loss of photosynthetic tissue, and presence of pests/pathogens) on the early life history of planted seedlings. We found effects of silvicultural treatments on damage and recovery rates that have not yet impacted the seedling survival rate. Repeated damage or the legacy effects of damage could very well reduce survival in the future and thus plantation success. The following discussion is an example of using transition rate and damage history to inform the assisted migration perspectives of eight tree species in the mixedwood forests of Eastern North America.

### 4.1 What is the Effect of Damage Frequency and Timing on Survival?

As hypothesized, earlier and recurring damage events increased mortality rates. Although this result is not surprising, its relatively similar effect size for species widely different in their requirements was unexpected, especially when considering responses to silvicultural treatments were species specific. We also observed temporally decreasing mortality rates for damaged seedlings (4 of 8 species) and increasing recovery rates (6 of 8 species), suggesting that seedlings are less likely to die after damage and surviving seedlings are more likely to recover from it as time passes. All damage types studied here are linked to some injury/loss of photosynthetic or structural tissues, either by abiotic or biotic agents. Recovery requires tissue replacement, which needs resources, mainly water, nitrogen, and carbon (Margolis and Brand 1990). Adequate access to the first two comes by a vigorous and well‐developed root system, which, once acquired, can devote carbon to aboveground growth that will subsidize carbon acquisition (Margolis and Brand 1990). Hence, we observed a negative effect of earlier damage and a time‐inhomogeneous process: seedling progressive establishment, that is, acquirements of an adequate access to site resources, improved their ability to recover from damage.

The lasting effect of earlier damage supports the presence of legacy effects, that is, effects that carry on beyond the duration of an event. For example, Ravn et al. (2024) demonstrated that the temperature in the previous growing season had stronger effects on height growth of balsam fir ( *Abies balsamea* ) than the current year temperature. They attributed this response to the effects of temperature on bud formation, reduced foliage production, and subsequent impact on stem and root growth. Similarly, damage as defined in our study could affect these physiological processes. This could generate a legacy effect if the damage is irreparable and/or increases vulnerability to similar damage or other stressors (Anderegg, Plavcova, et al. 2013); for example, Anderegg, Anderegg, and Berry (2013) presents a model of how precipitation deficit relates to physiological damage and trickles down to plant survival. Besides exceeding a physiological threshold, negative effects on growth could explain increased mortality for damaged seedlings in the following years; damaged seedlings with reduced growth rate could be so overcome by competing vegetation that they eventually die.

Higher recovery rates with more frequent and earlier damage are counter‐intuitive, but we suspect this result to be of a probabilistic origin. A seedling recorded ‘Damaged’ in year 1 has five “opportunities” in a six‐year survey to be later classified as ‘Healthy’, whereas a seedling first recorded ‘Damaged’ in year 5 only has one. Although we cannot maintain the high frequency of yearly survey, we do plan to individually monitor the seedlings in the future, and we will thus be able to assess further evolution of damage and recovery rates on the basis of early‐damage history. More generally, we suggest caution should be taken when considering very early snapshot surveys of plantation success, because of the time dependence of mortality, damage, and recovery rates and of legacy effects. Surveys realized 3 to 4 years after plantations have been shown to underestimate seedlings’ recovery, although they are the recommended timing in Finland (Luoranen et al. 2025).

A notable exception to the patterns described above is *Pinus strobus* ; for this species, earlier damage was not significantly associated with a decreasing mortality risk, and damage rate and mortality rates of damaged seedlings increased with time. We strongly suspect that this is related to infection by the fungus *Cronartium ribicola* that causes white pine blister rust, which was first noted in the study site in 2021, in the 4th survey. In our study, it was recorded as ‘Insects and diseases’ and is the most frequent damage for *Pinus strobus* , although closely followed by browsing (Table 2). *Cronartium ribicola* is an introduced fungus that causes mortality in seedlings and saplings, and infection is favored by the presence of *Ribes* spp., the alternative host (Maloy 1997) which is present at the study site. It is a reminder that some damaging agents have such a devastating impact on plants that they can drive entire population dynamics, no matter the level of establishment.

### 4.2 Can Silvicultural Treatments Modulate the Impact of Damage on Survival?

The cutting treatment had a strong effect on mortality rates, but also on damage and recovery rates. In our study, clearcuts and shelterwood cuts differed in temperature and light availability (2 times higher light availability in the clearcut; Dumais et al. 2025), but also in soil preparation, as clearcuts were scarified and shelterwood cuts were not. However, as results seem linked to species shade tolerance and because light is especially limiting at high latitudes (Lieffers et al. 1999; Messier et al. 1999), we propose that light availability is the principal factor that affected transition rates. The three species with the lowest shade tolerance had more favorable transition rates in the clearcut than in the shelterwood cut ( *Pinus strobus* , *Prunus serotina,* and *Pinus resinosa* ). *Pinus resinosa* especially presented lower damage and mortality rate in full light conditions; although the germination and early survival of this species benefit from cover, it reaches its full growth potential in full light conditions (Rudolf 1990). For *Prunus serotina* , hazard ratios were sometimes extremely large, which could be explained by the evolving light requirements of this species, which could generate more heterogeneity among seedlings. *Prunus serotina* establishes better in moderate light conditions, but requires full light to develop afterward (Marquis 1990; Verheyen et al. 2007; Cogliastro and Paquette 2012). High variability in this species’ responses could also be linked to variation in plantation stress, as our evaluation of photosynthetic capacity indicated a significant stress for these seedlings (Dumais et al. 2025). Similarly, Stone et al. (2025) found that shade tolerance (or gap affinity) explained interspecific variation in seedling demography, that is, survival and passage to the sapling stage. However, our results also show that, to a lesser extent, shade‐tolerant species performed better in the clearcut than in the shelterwood cut (see mortality rates of *Acer saccharum* and damage rates of *Acer saccharum* and *Picea rubens* ). This is also supported by growth, as all species presented greater diameter growth in the clearcut (Raymond et al. 2025); the shade‐tolerant *Picea rubens* also presented greater height growth in the clearcut. All seedlings were produced under full light conditions at the nursery and, therefore, are acclimated to full‐light conditions at the time of planting (Grossnickle 2000; Dumais and Prévost 2019); the physiological acclimation of foliage to local conditions can take more than a year. The decreasing damage rate and mortality rate of damaged *Picea rubens* support this process, as well as their evolving light‐saturated photosynthesis and specific leaf area, proxies of physio‐morphological acclimation (Dumais et al. 2025).

Alternatively, the more favorable transition rates for shade‐tolerant species in clearcuts could be attributed to the reduced competition in the former, because of scarification. Increasing mortality with time of healthy *Acer saccharum* and *Picea rubens* could also be attributed to the recolonization by competing vegetation, composed of fast‐growing species that increase in abundance and in height with time since treatments (e.g., Dumais and Prévost 2019). Nonetheless, this hypothesis is not supported by the sparse effects of competing vegetation removal on transition rates, which had no effect on *Picea rubens* and positive effects on the recovery rate of *Acer saccharum* . In fact, given that our results found both negative effects and positive effects of competitor presence (Table S3), this suggests the use of a neutral terminology, that is, neighboring plants. Neighboring plants’ presence reduced damage rates for *Thuja occidentalis* and *Prunus serotina* and increased recovery rates of *Acer saccharum* and *Quercus rubra* . It also paints a more nuanced portrait than our previous analyses, where removing competing vegetation improved conifers and *Prunus serotina* growth, without effects on 5‐year survival (Raymond et al. 2025). Neighboring plants could protect seedlings from abiotic or biotic perturbations. For example, tree seedlings growing within the canopy of shrubs benefit from a higher soil moisture and reduced environmental variability, with positive effects on survival and growth (Bertness and Callaway 1994; Gómez‐Aparicio et al. 2008; Filazzola and Lortie 2014). In the presence of herbivores, neighboring plants can act as alternative resources that decrease or increase the profitability of feeding patches, thus affecting seedlings' susceptibility to herbivores (Courant and Fortin 2010; Underwood et al. 2014; Champagne et al. 2020). In our experiment, effects of large herbivore exclusion were minimal, probably because most of the seedlings are still covered by snow during their first winters (mean annual snowfall of 230 cm), protecting them from ungulate herbivory in the prime season of browse consumption (see also Raymond et al. 2025). More evaluation on the mechanisms of facilitation in the context of assisted migration are required, as these effects could be leveraged to improve plantation success.

Shelterwood cutting systems have been proposed as solutions to acclimate planted seedlings and facilitate early survival, especially for assisted migration plantations (Paquette et al. 2006; Dumais et al. 2019; Royo et al. 2023). For example, shelterwood systems could reduce the risk of late frost damage by delaying budburst and increasing minimum temperatures (Langvall and Löfvenius 2002). Although we observed late frost damage on *Quercus rubra* , the analysis of damage history did not support this protective effect of partial cover as damage rates for five species (including *Quercus rubra* ) was higher in shelterwood than in the clearcut. Moreover, recovery rates are not higher in shelterwood, except for *Quercus rubra* and *Pinus strobus* . Less favorable conditions in the shelterwood cut compared to the clearcut can partially be explained by the processes described above, that is, appropriate regeneration niches and legacy effects of full light production in the nursery. We also suspect that we aggregated too many different types of damage in a single analysis (see Table 1). Moreover, we excluded from our analysis structural defects, such as the presence of multiple leaders, which could be associated with frost damage. For each species, the damage type recorded was diverse, and although some types have an identifiable cause (e.g., browsing), others are results of unidentifiable causes (e.g., dead leaders). Analyses with more specific damage, like frost damage, would probably provide a better understanding of damage risk and the capacity of partial cutting to buffer from perturbations and stresses.

### 4.3 Concluding Recommendations for Assisted Migration

Although providing adequate environmental conditions for quick seedling establishment is the basis of plantation silviculture, it is a message that bears repeating as we strive to adapt rapidly to changing conditions. Here, light availability appeared to be a critical factor for rapid establishment, which favored not only survival but also the ability of seedlings to withstand various forms of damage. Even for shade‐tolerant species, shelterwood cutting did not consistently reduce damage and improve recovery. Finer analyses, focusing on specific threats and assessing in detail the vegetation community surrounding the seedlings, are required. Moreover, longer‐term analyses should include assessment of structural defects that do not necessarily affect seedling survival but that affect wood production and quality. For example, winter desiccation and browsing can generate multiple leader stems in *Picea abies* , which are considered less desirable for wood production (Bergquist et al. 2003; Luoranen et al. 2006, 2025). However, multi‐trunk *Picea abies* can revert to a single‐trunk stage (Bergquist et al. 2003). Little is known of the actual capacity of seedlings to overcome early‐stage defects and produce high‐quality wood, especially for mid‐ or late‐successional deciduous species, some of which can have a weaker apical dominance, depending on environmental conditions (Millet et al. 1998).

We have not yet discussed the almost complete absence of difference in hazard rates among climate analogues. This result is in line with our ecophysiological study, where mid‐century and end‐of‐century analogues performed as well or even better than the current climate analogue (Dumais et al. 2025) and the limited differences in survival and growth among analogues (Raymond et al. 2025). These results bode well for our ability to translocate provenances and species, although this is still early days. Although translocation of trees is not new in silviculture, we need to innovate to displace planting stock that will not only be adapted to future climate conditions, but that will also be resistant or resilient to current and future damage.

Although the models presented here are a simplified representation of reality (i.e., grouping of damage types, simplification of experimental design) and their results are site‐specific (lack of random effects), multi‐state models helped us identify factors that may heighten the risk of individual stem mortality but have not yet impacted aggregate metrics of long‐term survival (e.g., browsing on *Thuja occidentalis* ). It also highlighted how ‘damage state’, in our capacity to assess it, can be transient and that we should be careful in equating damage with survival probability. This information will be used to inform further experimental tests and operational developments of assisted migration. In general, we propose that multi‐state model application is not limited to plantations and could be expanded to understand how treatments or stressors can impact tree properties, such as growth rate or wood quality. Analyses of damage and recovery, with any analytical framework, could provide early indicators of plantation success. These indicators would provide crucial information in the quick development of management plans required for the long‐term adaptation of managed forests.

## Author Contributions

**Emilie Champagne:** conceptualization (lead), data curation (lead), formal analysis (lead), methodology (equal), visualization (lead), writing – original draft (lead), writing – review and editing (lead). **Geneviève Picher:** formal analysis (supporting), writing – review and editing (supporting). **Daniel Dumais:** conceptualization (equal), methodology (equal), writing – review and editing (supporting). **Patricia Raymond:** conceptualization (equal), funding acquisition (lead), methodology (equal), project administration (lead), writing – review and editing (equal).

## Funding

This work was supported by Plan for a green economy, Gouvernement du Québec, 112959367. Direction de la recherche forestière, Ministère des Ressources naturelles et des Forêts, Gouvernement du Québec, 112332136.

## Conflicts of Interest

The authors declare no conflicts of interest.

## Supporting information


**Table S1:** Results of model selection comparing for each species multi‐state models with Treatments (Cutting, Competing vegetation, Cervid exclusion, and Analogues), Blocks and Year, and Treatments and Year to a null model with only Year as a covariate. Species are ordered on the basis of their general shade tolerance, from the most to the least tolerant. ΔAIC is calculated in reference to the ‘best’ model, with the lowest AIC. Models in bold are those for which results are reported.
**Table S2:** Results of model selection comparing for each species multi‐state models with either the Number of times in state ‘Damaged’ or the First year in state ‘Damaged’ as a covariate for two transitions (‘Damaged → Healthy’, ‘Damaged → Dead’) to a null model with only Year as a covariate. Species are ordered on the basis of their general shade tolerance, from the most to the least tolerant. ΔAIC is calculated in reference to the ‘best’ model, with the lowest AIC.
**Table S3:** Hazard ratios (HR) as predicted by multi‐state models to evaluate the effects of treatments on transition rates. Species are ordered on the basis of their general shade tolerance, from the most to the least tolerant. We present here the results from the best model as selected with a model selection approach (AIC and log‐ likelihood ratio test), comparing three models: (1) Treatments+Blocks+Year; (2) Treatments+Year; (3) Year (null model). When included in the best model, we do not present the HR related to block differences, as we were not interested in block effects per se. Estimates are presented with 95% confidence intervals in parenthesis and those in bold are statistically significant (CI does not include 1.0).
**Table S4:** Hazard ratios (HR) as predicted by multi‐state models to evaluate the effects of damage frequency and timing on transition rates. Species are ordered on the basis of their general shade tolerance, from the most to the least tolerant. We present here the results from two models: (1) Number of times in state ‘Damaged’+Year; (2) First year in state ‘Damaged’+Year. We do not present HR from the Year covariate, as the year effect was evaluated with a complete dataset. For these models, we used a subset of the dataset including only seedlings with at least 1 year in state ‘damaged’. Estimates are presented with 95% confidence intervals in parentheses, and those in bold are statistically significant (CI does not include 1.0).
**Figure S1:** Graphical assessment of models fit for Picea rubens.
**Figure S2:** Graphical assessment of models fit for Acer saccharum.
**Figure S3:** Graphical assessment of models fit for Thuja occidentalis.
**Figure S4:** Graphical assessment of models fit for Picea glauca.
**Figure S5:** Graphical assessment of models fit for Quercus rubra.
**Figure S6:** Graphical assessment of models fit for Pinus strobus.
**Figure S7:** Graphical assessment of models fit for Prunus serotina.
**Figure S8:** Graphical assessment of models fit for Pinus resinosa.

## Data Availability

Data and code are permanently archived in the Federated Research Data Repository (Champagne et al. 2026).

## References

[ece373496-bib-0001] Ameztegui, A. , and L. Coll . 2015. “Herbivory and Seedling Establishment in Pyrenean Forests: Influence of Micro‐And Meso‐Habitat Factors on Browsing Pressure.” Forest Ecology and Management 342: 103–111. 10.1016/j.foreco.2015.01.021.

[ece373496-bib-0002] Anderegg, L. D. , W. R. Anderegg , and J. A. Berry . 2013. “Not All Droughts Are Created Equal: Translating Meteorological Drought Into Woody Plant Mortality.” Tree Physiology 33: 701–712. 10.1093/treephys/tpt044. 23880634

[ece373496-bib-0003] Anderegg, W. R. , L. Plavcova , L. D. Anderegg , U. G. Hacke , J. A. Berry , and C. B. Field . 2013. “Drought's Legacy: Multiyear Hydraulic Deterioration Underlies Widespread Aspen Forest Die‐Off and Portends Increased Future Risk.” Global Change Biology 19: 1188–1196. 10.1111/gcb.12100. 23504895

[ece373496-bib-0004] Benomar, L. , J. Bousquet , M. Perron , J. Beaulieu , and M. Lamara . 2022. “Tree Maladaptation Under Mid‐Latitude Early Spring Warming and Late Cold Spell: Implications for Assisted Migration.” Frontiers in Plant Science 13: 920852. 10.3389/fpls.2022.920852. 35874013 PMC9298535

[ece373496-bib-0005] Bergquist, J. , R. Bergström , and A. Zakharenka . 2003. “Responses of Young Norway Spruce ( *Picea abies* ) to Winter Browsing by Roe Deer ( *Capreolus capreolus* ): Effects on Height Growth and Stem Morphology.” Scandinavian Journal of Forest Research 18: 368–376. 10.1080/0282758031005431.

[ece373496-bib-0006] Bertness, M. D. , and R. Callaway . 1994. “Positive Interactions in Communities.” Trends in Ecology & Evolution 9: 191–193. 10.1016/0169-5347(94)90088-4. 21236818

[ece373496-bib-0007] Burdett, A. N. 1990. “Physiological Processes in Plantation Establishment and the Development of Specifications for Forest Planting Stock.” Canadian Journal of Forest Research 20: 415–427. 10.1139/x90-059.

[ece373496-bib-0008] Burnham, K. P. , and D. R. Anderson . 2002. Model Selection and Multimodel Inference: A Practical Information‐Theoretic Approach. 2nd ed. Springer.

[ece373496-bib-0064] Champagne, E. , D. Dumais , P. Raymond , and G. Picher . 2026. “Seedlings Damage State From an Assisted Migration Trial in Temperate Mixedwood Forest.” Le Dépôt fédéré de données de recherche. 10.20383/103.01362.

[ece373496-bib-0009] Champagne, E. , B. D. Moore , S. D. Côté , and J.‐P. Tremblay . 2020. “Intraspecific Variation in Nutritional Traits of Neighbouring Plants Generates a Continuum of Associational Effects.” Journal of Vegetation Science 31: 920–933. 10.1111/jvs.12914.

[ece373496-bib-0010] Champagne, E. , A. A. Royo , J.‐P. Tremblay , and P. Raymond . 2021. “Tree Assisted Migration in a Browsed Landscape: Can We Predict Susceptibility to Herbivores?” Forest Ecology and Management 498: 119576. 10.1016/j.foreco.2021.119576.

[ece373496-bib-0011] Champagne, E. , R. Turgeon , A. D. Munson , and P. Raymond . 2021. “Seedling Response to Simulated Browsing and Reduced Water Availability: Insights for Assisted Migration Plantations.” Forests 12: 1396. 10.3390/f12101396.

[ece373496-bib-0012] Chang, H. , J. An , Y. Roh , and Y. Son . 2020. “Experimental Warming and Drought Treatments Reduce Physiological Activities and Increase Mortality of *Pinus koraiensis* Seedlings.” Plant Ecology 221: 515–527. 10.1007/s11258-020-01030-3.

[ece373496-bib-0013] Clark, P. W. , A. W. D'Amato , B. J. Palik , et al. 2023. “A Lack of Ecological Diversity in Forest Nurseries Limits the Achievement of Tree‐Planting Objectives in Response to Global Change.” Bioscience 73: 575–586. 10.1093/biosci/biad049.

[ece373496-bib-0014] Cogliastro, A. , and A. Paquette . 2012. “Thinning Effect on Light Regime and Growth of Underplanted Red Oak and Black Cherry in Post‐Agricultural Forests of South‐Eastern Canada.” New Forests 43: 941–954. 10.1007/s11056-012-9329-5.

[ece373496-bib-0015] Courant, S. , and D. Fortin . 2010. “Foraging Decisions of Bison for Rapid Energy Gains Can Explain the Relative Risk to Neighboring Plants in Complex Swards.” Ecology 91: 1841–1849. 10.1890/09-1226.1. 20583724

[ece373496-bib-0016] Dumais, D. , C. Larouche , P. Raymond , S. Bédard , and M.‐C. Lambert . 2019. “Survival and Growth Dynamics of Red Spruce Seedlings Planted Under Different Forest Cover Densities and Types.” New Forests 50: 573–592. 10.1007/s11056-018-9680-2.

[ece373496-bib-0018] Dumais, D. , and M. Prévost . 2019. “Nine‐Year Physiology, Nutrition and Morphological Development of *Picea glauca* Reintroduced by Planting in a High‐Graded Yellow Birch–Conifer Stand.” Scandinavian Journal of Forest Research 34: 656–666. 10.1080/02827581.2019.1656771.

[ece373496-bib-0019] Dumais, D. , P. Raymond , and E. Champagne . 2025. “Translocated Southern Seedlings Perform as Well as Local Provenances: Insights From an Ecophysiological Monitoring Under Varying Cutting Modalities.” New Forests 56: 20. 10.1007/s11056-024-10089-z.

[ece373496-bib-0021] Eulenburg, C. , S. Mahner , L. Woelber , and K. Wegscheider . 2015. “A Systematic Model Specification Procedure for an Illness‐Death Model Without Recovery.” PLoS One 10: e0123489. 10.1371/journal.pone.0123489. 25874628 PMC4395319

[ece373496-bib-0022] Filazzola, A. , and C. J. Lortie . 2014. “A Systematic Review and Conceptual Framework for the Mechanistic Pathways of Nurse Plants.” Global Ecology and Biogeography 23: 1335–1345. 10.1111/geb.12202.

[ece373496-bib-0023] Gentleman, R. C. , J. F. Lawless , J. C. Lindsey , and P. Yan . 1994. “Multi‐State Markov Models for Analysing Incomplete Disease History Data With Illustrations for HIV Disease.” Statistics in Medicine 13: 805–821. 10.1002/sim.4780130803. 7914028

[ece373496-bib-0024] Gómez‐Aparicio, L. , R. Zamora , J. Castro , and J. A. Hódar . 2008. “Facilitation of Tree Saplings by Nurse Plants: Microhabitat Amelioration or Protection Against Herbivores?” Journal of Vegetation Science 19: 161–172. 10.3170/2008-8-18347.

[ece373496-bib-0025] Groha, S. , S. M. Schmon , and A. Gusev . 2021. “A General Framework for Survival Analysis and Multi‐State Modelling.” arXiv:2006.04893, 10.48550/arXiv.2006.04893.

[ece373496-bib-0026] Grossnickle, S. C. 2000. Ecophysiology of Northern Spruce Species. The Performance of Planted Seedlings. NRC Research Press.

[ece373496-bib-0027] Grossnickle, S. C. 2005. “Importance of Root Growth in Overcoming Planting Stress.” New Forests 30: 273–294. 10.1007/s11056-004-8303-2.

[ece373496-bib-0028] Guo, J. , Y. Yang , G. Wang , L. Yang , and X. Sun . 2010. “Ecophysiological Responses of *Abies fabri* Seedlings to Drought Stress and Nitrogen Supply.” Physiologia Plantarum 139: 335–347. 10.1111/j.1399-3054.2010.01370.x. 20230480

[ece373496-bib-0030] Jackson, C. H. 2011. “Multi‐State Models for Panel Data: The Msm Package for R.” Journal of Statistical Software 38: 1–29. 10.18637/jss.v038.i08.

[ece373496-bib-0029] Jackson, C. H. 2024. “Multi‐State Modelling With R: The MSM Package. Version 1.8.2.”

[ece373496-bib-0031] Jackson, C. H. 2025. “Multi‐State Modelling With MSM: A Practical Course.”

[ece373496-bib-0032] Langvall, O. , and M. O. Löfvenius . 2002. “Effect of Shelterwood Density on Nocturnal Near‐Ground Temperature, Frost Injury Risk and Budburst Date of Norway Spruce.” Forest Ecology and Management 168: 149–161. 10.1016/S0378-1127(01)00754-X.

[ece373496-bib-0033] Lieffers, V. J. , C. Messier , K. J. Stadt , F. Gendron , and P. G. Comeau . 1999. “Predicting and Managing Light in the Understory of Boreal Forests.” Canadian Journal of Forest Research 29: 796–811. 10.1139/x98-165.

[ece373496-bib-0034] Luoranen, J. , R. Rikala , K. Konttinen , and H. Smolander . 2006. “Summer Planting of *Picea abies* Container‐Grown Seedlings: Effects of Planting Date on Survival, Height Growth and Root Egress.” Forest Ecology and Management 237: 534–544. 10.1016/j.foreco.2006.09.073.

[ece373496-bib-0035] Luoranen, J. , A. Salmivaara , and J. Miina . 2025. “Recovery of Planted Spruce Seedlings From Abiotic Damage Caused by Exceptional Weather Conditions in the Boreal Forest: Identification of Risks Associated With Site Selection and Regeneration Practices.” Forest Ecology and Management 585: 122620. 10.1016/j.foreco.2025.122620.

[ece373496-bib-0036] Maloy, O. C. 1997. “White Pine Blister Rust Control in North America: A Case History.” Annual Review of Phytopathology 35: 87–109. 10.1146/annurev.phyto.35.1.87 15012516

[ece373496-bib-0037] Margolis, H. A. , and D. G. Brand . 1990. “An Ecophysiological Basis for Understanding Plantation Establishment.” Canadian Journal of Forest Research 20: 375–390. 10.1139/x90-056.

[ece373496-bib-0038] Marquis, D. A. 1990. “Black Cherry.” In Silvics of North America. Volume 2: Hardwoods, edited by R. M. Burns and B. H. Honkala . United States Department of Agriculture (USDA), Forest Service.

[ece373496-bib-0039] Mazerolle, M. J. 2006. “Improving Data Analysis in Herpetology: Using Akaike's Information Criterion (AIC) to Assess the Strength of Biological Hypotheses.” Amphibia‐Reptilia 27: 169–180. 10.1163/156853806777239922.

[ece373496-bib-0040] Messier, C. , J. Bauhus , R. Sousa‐Silva , et al. 2021. “For the Sake of Resilience and Multifunctionality, Let's Diversify Planted Forests!” Conservation Letters 15: e12829. 10.1111/conl.12829.

[ece373496-bib-0041] Messier, C. , R. Doucet , J.‐C. Ruel , Y. Claveau , C. Kelly , and M. J. Lechowicz . 1999. “Functional Ecology of Advance Regeneration in Relation to Light in Boreal Forests.” Canadian Journal of Forest Research 29: 812–823. 10.1139/x99-070.

[ece373496-bib-0042] Millar, C. I. , N. L. Stephenson , and S. L. Stephens . 2007. “Climate Change and Forests of the Future: Managing in the Face of Uncertainty.” Ecological Applications 17: 2145–2151. 10.1890/06-1715.1. 18213958

[ece373496-bib-0043] Millet, J. , A. Bouchard , and C. Édelin . 1998. “Plagiotropic Architectural Development of Four Tree Species of the Temperate Forest.” Canadian Journal of Botany 76: 2100–2118. 10.1139/b98-174.

[ece373496-bib-0044] Ministère des Ressources naturelles , ed. 2013. Le Guide Sylvicole du Québec, Tome 1, Les Fondements Biologiques de la Sylviculture. Les Publications du Québec.

[ece373496-bib-0045] Mura, C. , V. Butto , R. Silvestro , et al. 2022. “The Early Bud Gets the Cold: Diverging Spring Phenology Drives Exposure to Late Frost in a *Picea mariana* [(Mill.) BSP] Common Garden.” Physiologia Plantarum 174: e13798. 10.1111/ppl.13798. 36251716

[ece373496-bib-0046] Nagel, L. M. , B. J. Palik , M. A. Battaglia , et al. 2017. “Adaptive Silviculture for Climate Change: A National Experiment in Manager‐Scientist Partnerships to Apply an Adaptation Framework.” Journal of Forestry 115: 167–178. 10.5849/jof.16-039.

[ece373496-bib-0047] O'Reilly‐Wapstra, J. M. , B. D. Moore , M. Brewer , et al. 2014. “ *Pinus sylvestris* Sapling Growth and Recovery From Mammalian Browsing.” Forest Ecology and Management 325: 18–25. 10.1016/j.foreco.2014.03.038.

[ece373496-bib-0048] Paquette, A. , A. Bouchard , and A. Cogliastro . 2006. “Successful Under‐Planting of Red Oak and Black Cherry in Early‐Successional Deciduous Shelterwoods of North America.” Annals of Forest Science 63: 823–831. 10.1051/forest:2006065.

[ece373496-bib-0049] Pedlar, J. H. , D. W. McKenney , I. Aubin , et al. 2012. “Placing Forestry in the Assisted Migration Debate.” Bioscience 62: 835–842. 10.1525/bio.2012.62.9.10.

[ece373496-bib-0050] R Core Team . 2024. “R: A Language and Environment for Statistical Computing.” R Foundation for Statistical Computing, Vienna, Austria.

[ece373496-bib-0051] Ravn, J. , A. R. Taylor , M. B. Lavigne , and L. D'Orangeville . 2024. “Local Adaptation of Balsam Fir Seedlings Improves Growth Resilience to Heat Stress.” Canadian Journal of Forest Research 54: 331–343. 10.1139/cjfr-2023-0128.

[ece373496-bib-0052] Raymond, P. , E. Champagne , D. Dumais , et al. 2025. “Moving Up North: How Do Translocated Seedlings Thrive in Mixed‐Species Plantings at the Boreal‐Temperate Interface?” Forest Ecology and Management 597: 123179.

[ece373496-bib-0055] Redick, C. H. , J. R. McKenna , D. E. Carlson , M. A. Jenkins , and D. F. Jacobs . 2020. “Silviculture at Establishment of Hardwood Plantations is Relatively Ineffective in the Presence of Deer Browsing.” Forest Ecology and Management 474: 118339. 10.1016/j.foreco.2020.118339.

[ece373496-bib-0056] Royo, A. A. , P. Raymond , C. C. Kern , et al. 2023. “Desired REgeneration Through Assisted Migration (DREAM): Implementing a Research Framework for Climate‐Adaptive Silviculture.” Forest Ecology and Management 546: 121298. 10.1016/j.foreco.2023.121298.

[ece373496-bib-0057] Rudolf, P. O. 1990. “Red Pine.” In Silvics of North America. Volume 1: Conifers, edited by R. M. Burns and B. H. Honkala . United States Department of Agriculture (USDA), Forest Service.

[ece373496-bib-0058] Saucier, J.‐P. , A. Robitaille , and P. Grondin . 2009. “Cadre Bioclimatique du Québec.” In Manuel de Foresterie, Deuxième Édition, edited by R. Doucet and M. Côté , 186–205. Éditions Multimonde.

[ece373496-bib-0059] Stone, I. , J. Mintz , C. J. Garnica‐Díaz , et al. 2025. “Seedling Passage Times in Gaps and Closed Canopies Reveal Decades of Understory Persistence in a New England Forest.” Ecosphere 16: e70273. 10.1002/ecs2.70273.

[ece373496-bib-0060] Tear, E. C. , K. O. Higginbotham , and J. M. Mayo . 1982. “Effects of Drying Soils on Survival o Young *Picea glauca* Seedlings.” Canadian Journal of Forest Research 12: 1005–1009. 10.1139/x82-144.

[ece373496-bib-0061] Thiffault, N. , P. Raymond , J.‐M. Lussier , et al. 2021. “Adaptive Silviculture for Climate Change: From Concepts to Reality Report on a Symposium Held at Carrefour Forêts 2019.” Forestry Chronicle 97: 13–27. 10.5558/tfc2021-004.

[ece373496-bib-0062] Underwood, N. , B. D. Inouye , and P. A. Hambäck . 2014. “A Conceptual Framework for Associational Effects: When Do Neighbors Matter and How Would We Know?” Quarterly Review of Biology 89: 1–19. 10.1086/674991. 24672901

[ece373496-bib-0063] Verheyen, K. , M. Vanhellemont , T. Stock , and M. Hermy . 2007. “Predicting Patterns of Invasion by Black Cherry ( *Prunus serotina* Ehrh.) in Flanders (Belgium) and Its Impact on the Forest Understorey Community.” Diversity and Distributions 13: 487–497. 10.1111/j.1472-4642.2007.00334.x.

