# Supplementary material for: Will It Survive? Evaluating the Effects of Damage and Silviculture on Tree Seedling Survival Using Multi‐State Models
Source: Ecol Evol. 2026 May 6;16(5):e73496. doi: 10.1002/ece3.73496 (PMC13147155; doi:10.1002/ece3.73496)
Supplement: Supplementary file 1 — Table S1: Results of model selection comparing for each species multi‐state models with Treatments (Cutting, Competing vegetation, Cervid exclusion, and Analogues), Blocks and Year, and Treatments and Year to a null model with only Year as a covariate. Species are ordered on the basis of their general shade tolerance, from the most to the least tolerant. ΔAIC is calculated in reference to the ‘best’ model, with the lowest AIC. Models in bold are those for which results are reported. Table S2: Results of model selection comparing for each species multi‐state models with either the Number of times in state ‘Damaged’ or the First year in state ‘Damaged’ as a covariate for two transitions (‘Damaged → Healthy’, ‘Damaged → Dead’) to a null model with only Year as a covariate. Species are ordered on the basis of their general shade tolerance, from the most to the least tolerant. ΔAIC is calculated in reference to the ‘best’ model, with the lowest AIC. Table S3: Hazard ratios (HR) as predicted by multi‐state models to evaluate the effects of treatments on transition rates. Species are ordered on the basis of their general shade tolerance, from the most to the least tolerant. We present here the results from the best model as selected with a model selection approach (AIC and log‐ likelihood ratio test), comparing three models: (1) Treatments+Blocks+Year; (2) Treatments+Year; (3) Year (null model). When included in the best model, we do not present the HR related to block differences, as we were not interested in block effects per se. Estimates are presented with 95% confidence intervals in parenthesis and those in bold are statistically significant (CI does not include 1.0). Table S4: Hazard ratios (HR) as predicted by multi‐state models to evaluate the effects of damage frequency and timing on transition rates. Species are ordered on the basis of their general shade tolerance, from the most to the least tolerant. We present here the results from two models: (1) Number of ti [file ECE3-16-e73496-s001.pdf]

Supporting information

Manuscript title: Will it survive? Evaluating the effects of damage and silviculture on tree seedling survival using multi-state models

Authors: Emilie Champagne*, Daniel Dumais, Geneviève Picher, and Patricia Raymond

Journal : Ecology and Evolution

Table S1. Results of model selection comparing for each species multi-state models with Treatments (*Cutting*, *Competing vegetation*, *Cervid exclusion*, *Analogues*), *Blocks* and *Year*, and Treatments and *Year* to a null model with only *Year* as a covariate. Species are ordered based on their general shade tolerance, from the most to the least tolerant. ΔAIC are calculated in reference to the 'best' model, with the lowest AIC. Models in bold are those for which results are reported.

**Species**	**Models**	**Number of parameters**	**AIC**	**ΔAIC**
Picea rubens	**Treatments + Year + Blocks**	40	6704	
	Treatments + Year	28	6748	45
	Year	8	6830	126
Acer saccharum	**Treatments + Year + Blocks**	40	7323	
	Treatments + Year	28	7382	59
	Year	8	7509	186
Thuja occidentalis	**Treatments + Year + Blocks**	40	6386	
	Treatments + Year	28	6398	11
	Year	8	6518	132
Picea glauca	Treatments + Year + Blocks	40	6138	
	**Treatments + Year**	28	6143	5
	Year	8	6209	71
Quercus rubra	**Treatments + Year**	28	7084	
	Year	8	7114	30
	Treatments + Year + Blocks	Did not converge		
Pinus strobus	**Treatments + Year + Blocks**	40	7666	
	Treatments + Year	28	7684	17
	Year	8	7766	99
Prunus serotina	**Treatments + Year + Blocks**	40	7124	
	Treatments + Year	28	7173	49
	Year	8	7212	88
Pinus resinosa	**Treatments + Year**	28	5799	
	Treatments + Year + Blocks	40	5812	13
	Year	8	5848	36

Table S2. Results of model selection comparing for each species multi-state models with either the *Number of times in state 'Damaged'* or the *First year in state 'Damaged'* as a covariate for two transitions ('Damaged → Healthy', 'Damaged → Dead') to a null model with only *Year* as a covariate. Species are ordered based on their general shade tolerance, from the most to the least tolerant. ΔAIC are calculated in reference to the 'best' model, with the lowest AIC.

**Species**	**Models**	**Number of parameters**	**AIC**	**ΔAIC**
Picea rubens	First year + Year	10	4854	
	Number of times + Year	10	4970	116
	Year	8	4985	131
Acer saccharum	First year + Year	10	6224	
	Number of times + Year	10	6285	61
	Year	8	6323	99
Thuja occidentalis	First year + Year	10	4760	
	Number of times + Year	10	4815	55
	Year	8	4855	95
Picea glauca	First year + Year	10	4194	
	Number of times + Year	10	4289	95
	Year	8	4342	149
Quercus rubra	First year + Year	10	6071	
	Number of times + Year	10	6094	23
	Year	8	6163	92
Pinus strobus	First year + Year	10	5968	
	Number of times + Year	10	6255	287
	Year	8	6293	325
Prunus serotina	First year + Year	10	5515	
	Number of times + Year	10	5614	99
	Year	8	5666	151
Pinus resinosa	First year + Year	10	3802	
	Number of times + Year	10	3847	45
	Year	8	3954	107

Table S3 Hazard ratios (HR) as predicted by multi-state models to evaluate the effects of treatments on transition rates. Species are ordered based on their general shade tolerance, from the most to the least tolerant. We present here the results from the best model as selected with a model selection approach (AIC and log-likelihood ratio test), comparing three models: 1) Treatments + Blocks + Year; 2) Treatments + Year; 3) Year (null model). When included in the best model, we do not present the HR related to block differences as we were not interested in block effects per se. Estimates are presented with 95% confidence intervals in parenthesis and those in bold are statistically significant (CI does not include 1.0).

**Treatment**	**Interpretation**	**Transition**	**Picea rubens**	**Acer saccharum**	**Thuja occidentalis**	**Picea glauca**	**Quercus rubra**	**Pinus strobus**	**Prunus serotina**	**Pinus resinosa**
Year	Continuous and progressive<br>< 1 = decrease with time<br>> 1 = increase	H. → Dam.	**0.71 (0.66, 0.77)**	1.2 (1.0, 1.6)	**0.92 (0.85, 0.99)**	1.1 (1.0, 1.2)	1.1 (0.9, 1.3)	**1.5 (1.3, 1.7)**	2.2 (1.0, 5.0)	1.0 (0.9, 1.1)
		H. → Dead	**1.8 (1.2, 2.8)**	**4.2 (1.4, 12.1)**	**0.06 (0.00, 0.83)**	1.4 (0.9, 2.2)	0.1 (0.0, 2.5)	0.03 (0.00, 1.64)	**0.6 (0.4, 0.9)**	**0.2 (0.1, 0.4)**
		Dam. → H.	**1.2 (1.1, 1.4)**	**1.9 (1.4, 2.4)**	1.1 (1.0, 1.2)	**1.2 (1.1, 1.4)**	**1.8 (1.5, 2.3)**	1.1 (1.0, 1.3)	**2.9 (1.3, 6.4)**	**1.2 (1.1, 1.3)**
		Dam. → Dead	**0.5 (0.3, 0.7)**	**0.6 (0.4, 0.8)**	**0.6 (0.5, 0.7)**	0.8 (0.7, 1.0)	1.4 (1.0, 1.9)	**1.3 (1.1, 1.6)**	**0.4 (0.3, 0.5)**	0.8 (0.7, 1.0)
Overstory cutting	<1 =<br>shelter < clearcut<br>>1 =<br>shelter > clearcut	H. → Dam.	**2.1 (1.7, 2.6)**	**0.4 (0.2, 0.7)**	0.9 (0.7, 1.1)	**1.3 (1.1, 1.6)**	**1.8 (1.1, 3.1)**	**6.2 (2.9, 13.5)**	54 (0, 6258)	**1.4 (1.1, 1.8)**
		H. → Dead	0.4 (0.1, 1.0)	**3.1 (1.2, 7.7)**	172 (0, $3x10^5$)	1.7 (0.5, 5.4)	0.9 (0.5, 1.8)	**3.4 (1.5, 7.7)**	188 (0, $4x10^6$)	**2.9 (1.4, 6.0)**
		Dam. → H.	1.1 (0.8, 1.4)	**0.4 (0.2, 0.9)**	**0.7 (0.5, 0.8)**	**0.7 (0.5, 0.8)**	**2.6 (1.5, 4.6)**	**5.0 (2.2, 11.3)**	48 (0, 5598)	0.8 (0.6, 1.0)
		Dam. → Dead	1.2 (0.8, 1.8)	0.8 (0.6, 1.3)	1.3 (0.8, 1.9)	**0.5 (0.3, 0.9)**	0.8 (0.5, 1.5)	0.8 (0.6, 1.2)	**0.6 (0.4, 0.8)**	**1.7 (1.1, 2.7)**
Cervid exclusion	<1 =<br>no fence (1) < fence<br>>1 =<br>no fence > fence	H. → Dam.	1.0 (0.8, 1.3)	0.7 (0.5, 1.1)	**1.7 (1.4, 2.1)**	1.0 (0.8, 1.2)	0.7 (0.5, 1.1)	1.0 (0.7, 1.3)	**0.1 (0.0, 0.6)**	1.0 (0.8, 1.2)
		H. → Dead	0.4 (0.2, 1.0)	0.5 (0.2, 1.1)	0.3 (0.1, 1.0)	0.2 (0.0, 1.2)	0.8 (0.4, 1.6)	1.9 (0.9, 4.2)	0.1 (0.0, 2.5)	1.0 (0.5, 2.1)
		Dam. → H.	1.1 (0.9, 1.4)	**0.6 (0.3, 0.9)**	0.8 (0.6, 1.1)	1.1 (0.9, 1.4)	0.6 (0.4, 1.0)	1.1 (0.8, 1.6)	**0.1 (0.0, 0.5)**	1.2 (0.9, 1.6)
		Dam. → Dead	0.8 (0.5, 1.1)	1.3 (0.9, 1.9)	1.3 (0.8, 1.9)	1.1 (0.7, 1.7)	0.8 (0.4, 1.5)	1.0 (0.7, 1.4)	1.3 (1.0, 1.9)	0.7 (0.5, 1.1)
Competing vegetation	<1 =<br>present (1) < removed<br>>1 =<br>present > removed	H. → Dam.	0.9 (0.7, 1.1)	1.9 (0.8, 4.6)	**0.7 (0.6, 0.9)**	0.9 (0.7, 1.0)	**2.6 (1.5, 4.7)**	0.9 (0.7, 1.2)	**0.2 (0.0, 0.9)**	0.9 (0.7, 1.1)
		H. → Dead	0.8 (0.4, 1.7)	1.9 (0.9, 4.0)	1.9 (0.7, 5.1)	38 (0, 19525)	2.2 (0.8, 5.9)	1.0 (0.5, 2.1)	0.5 (0.2, 1.5)	0.7 (0.3, 1.4)
		Dam. → H.	0.8 (0.6, 1.0)	**2.7 (1.1, 6.7)**	0.9 (0.7, 1.2)	**0.7 (0.6, 0.9)**	**2.7 (1.5, 5.0)**	0.9 (0.7, 1.3)	0.2 (0.1, 1.1)	0.8 (0.6, 1.1)

		Dam. → Dead	1.5 (1.0, 2.2)	1.1 (0.8, 1.6)	1.3 (0.8, 1.9)	0.8 (0.5, 1.4)	0.8 (0.4, 1.6)	1.0 (0.7, 1.3)	**1.4 (1.1, 2.0)**	1.5 (1.0, 2.3)

Treatment	Interpretation	Transition	Picea rubens	Acer saccharum	Thuja occidentalis	Picea glauca	Quercus rubra	Pinus strobus	Prunus serotina	Pinus resinosa
Analogue	<1 =<br>M-cent < Current<br>>1 =<br>M-cent > Current	H. → Dam.	1.3 (1.0, 1.7)	1.3 (0.7, 2.2)	1.0 (0.7, 1.3)	1.2 (1.0, 1.6)	**2.5 (1.2, 5.4)**	1.4 (1.0, 2.0)	2.3 (0.8, 6.3)	1.2 (0.9, 1.5)
		H. → Dead	1.3 (0.6, 2.8)	0.5 (0.2, 1.2)	2.0 (0.6, 7.3)	1.4 (0.5, 3.8)	1.9 (0.7, 5.1)	0.5 (0.2, 1.4)	11 (0, $3x10^{5}$)	0.5 (0.2, 1.7)
		Dam. → H.	1.0 (0.7, 1.3)	1.1 (0.6, 2.1)	1.3 (1.0, 1.8)	1.0 (0.8, 1.4)	**2.6 (1.2, 5.8)**	1.2 (0.8, 1.8)	1.9 (0.7, 5.4)	0.9 (0.7, 1.3)
		Dam. → Dead	0.9 (0.6, 1.5)	0.6 (0.4, 1.1)	1.4 (0.8, 2.3)	0.7 (0.4, 1.4)	0.8 (0.4, 1.6)	0.8 (0.6, 1.3)	**0.4 (0.3, 0.6)**	1.0 (0.6, 1.7)
	<1 =<br>E-cent < Current<br>>1 =<br>E-cent > Current	H. → Dam.	1.1 (0.9, 1.5)	4.4 (2.2, 8.9)	0.8 (0.7, 1.0)	1.3 (1.0, 1.6)	1.7 (1.1, 2.6)	1.1 (0.8, 1.6)	58 (0, $1x10^{4}$)	0.9 (0.7, 1.2)
		H. → Dead	0.8 (0.3, 2.0)	1.2 (0.6, 2.6)	3.1 (0.9, 10.2)	0.5 (0.1, 2.9)	2.3 (0.9, 5.8)	0.9 (0.4, 2.0)	47 (0, $1x10^{5}$)	1.4 (0.7, 3.1)
		Dam. → H.	0.9 (0.7, 1.2)	**2.3 (1.1, 4.9)**	0.8 (0.6, 1.0)	0.9 (0.7, 1.3)	1.5 (1.0, 2.3)	0.8 (0.5, 1.2)	45 (0, $8 x10^{3}$)	0.8 (0.6, 1.2)
		Dam. → Dead	1.0 (0.6, 1.6)	0.7 (0.5, 1.2)	1.5 (0.9, 2.4)	1.0 (0.6, 1.7)	0.5 (0.2, 1.3)	0.9 (0.6, 1.4)	0.7 (0.5, 1.0)	0.9 (0.5, 1.5)

Table S4 Hazard ratios (HR) as predicted by multi-state models to evaluate the effects of damage frequency and timing on transition rates. Species are ordered based on their general shade tolerance, from the most to the least tolerant. We present here the results from two models: 1) Number of times in state ‘Damaged’ + Year; 2) First year in state ‘Damaged’ + Year. We do not present HR from the Year covariate, as year effect was evaluated with a complete dataset. For these models, we used a subset of the dataset including only seedlings with at least one year in state ‘damaged’. Estimates are presented with 95% confidence intervals in parenthesis and those in bold are statistically significant (CI does not include 1.0).

**Treatment**	**Interpretation**	**Transition**	**Picea rubens**	**Acer saccharum**	**Thuja occidentalis**	**Picea glauca**	**Quercus rubra**	**Pinus strobus**	**Prunus serotina**	**Pinus resinosa**
Number of times in ‘damaged’	Continuous and progressive<br>< 1 = decrease with variable<br>> 1 = increase	Dam. → H.	**1.2 (1.1, 1.3)**	**1.2 (1.1, 1.3)**	**1.4 (1.2, 1.5)**	**1.5 (1.3. 1.7)**	**1.4 (1.3, 1.5)**	**1.4 (1.2, 1.5)**	**1.4 (1.3, 1.6)**	**1.9 (1.6, 2.2)**
		Dam. → Dead	**2.0 (1.1, 3.4)**	**2.2 (1.6, 2.9)**	**2.0 (1.5, 2.8)**	**2.5 (1.8, 3.4)**	**3.9 (2.3, 6.7)**	**1.5 (1.2, 1.8)**	**3.3 (1.6, 6.7)**	**4.6 (2.9, 7.5)**
First year in ‘damaged’	Continuous and constant<br>< 1 = decrease with variable<br>> 1 = increase	Dam. → H.	**1.4 (1.3, 1.5)**	**1.3 (1.2, 1.4)**	1**.4 (1.3, 1.5)**	**1.6 (1.5, 1.7)**	**1.4 (1.3, 1.5)**	**1.7 (1.6, 1.8)**	**1.4 (1.3, 1.5)**	**1.5 (1.4, 1.7)**
		Dam. → Dead	**0.4 (0.2, 0.8)**	**0.6 (0.5, 0.8)**	**0.7 (0.5, 0.9)**	**0.6 (0.5, 0.8)**	**0.5 (0.3, 0.9)**	0.9 (0.7, 1.0)	**0.4 (0.2, 0.8)**	**0.4 (0.3, 0.5)**

For all following figures: Visual test proposed by Gentleman et al. (1994), based on code provided by Joseph Rickert (https://rviews.rstudio.com/2023/04/19/multistate-models-for-medical-applications/), presenting the observed and expected values of prevalence. Expected values are extracted from the models presented in the manuscript. For each species, three figures are presented, one for each multi-state model. In each figure, three graphs present the progression in prevalence of each state (‘Healthy’, ‘Damaged’ and ‘Dead’) through time.

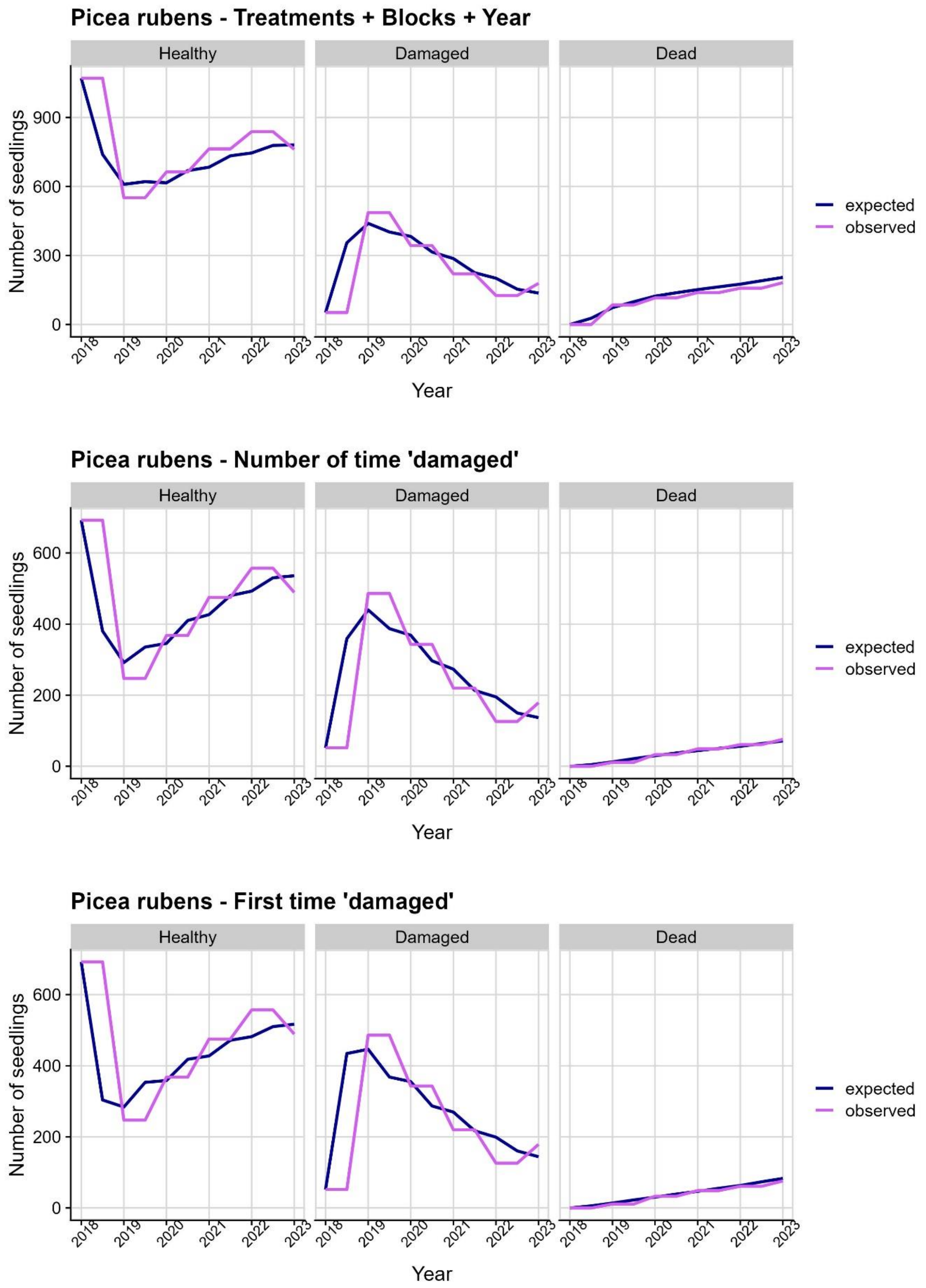

Figure S1. Graphical assessment of models fit for *Picea rubens*.

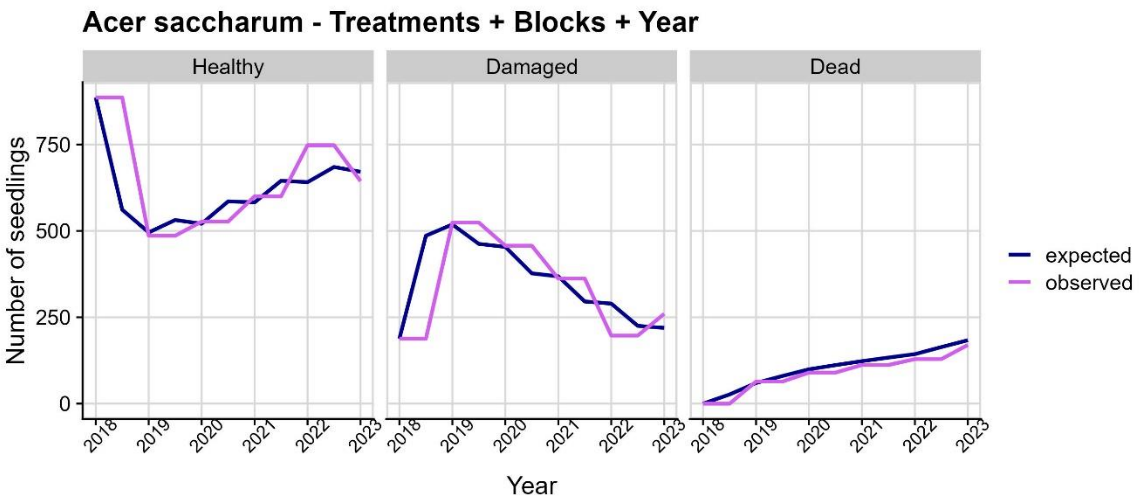

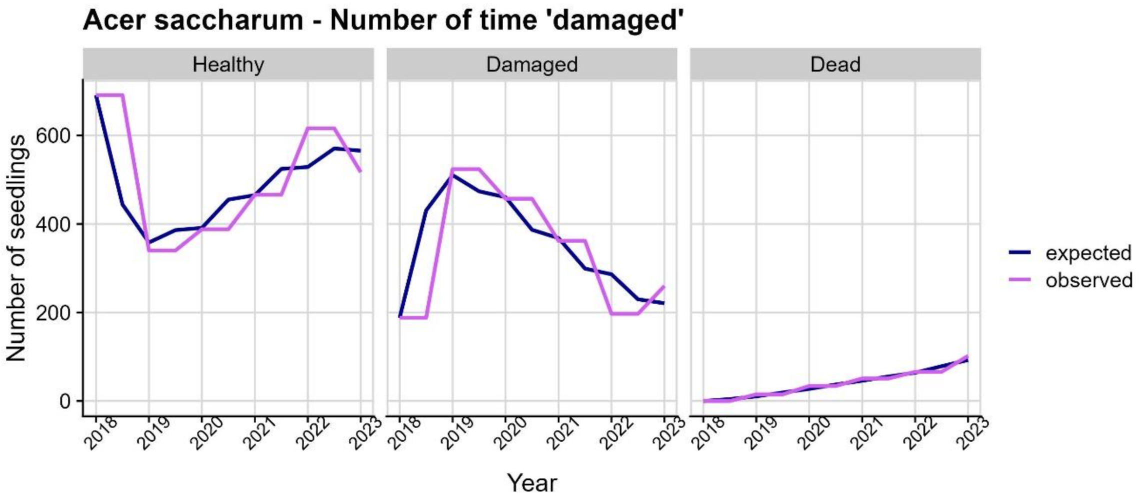

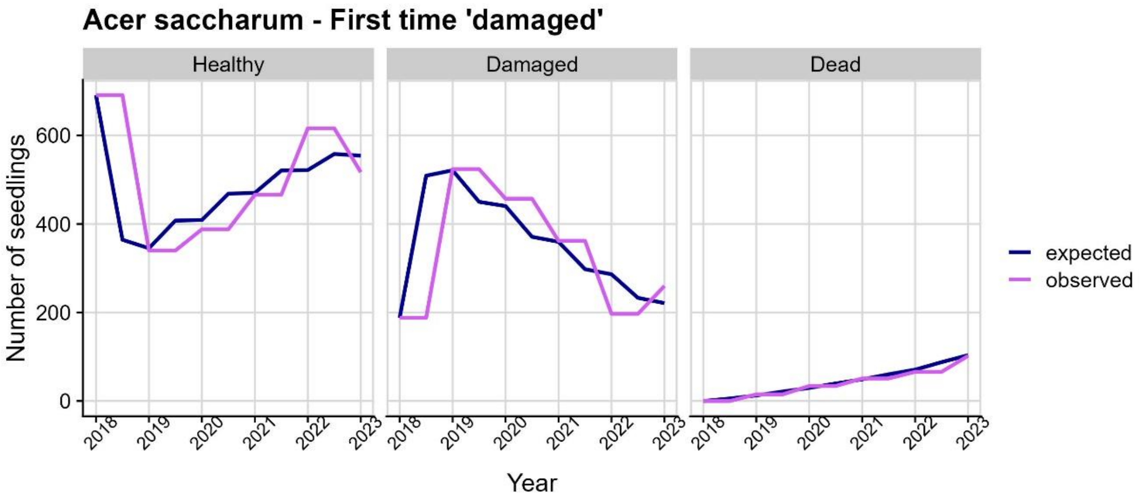

Figure S2. Graphical assessment of models fit for *Acer saccharum*.

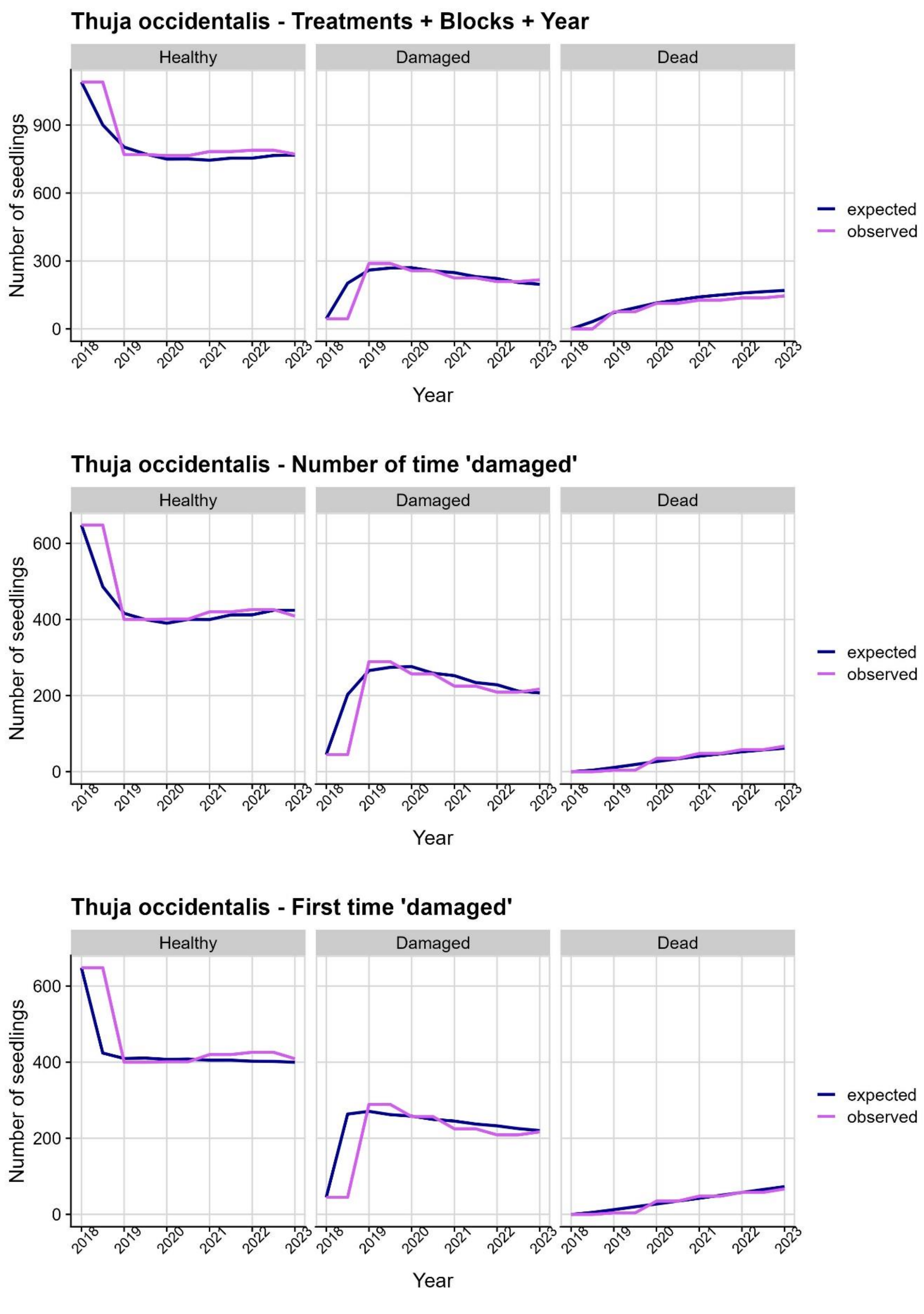

Figure S3. Graphical assessment of models fit for *Thuja occidentalis*.

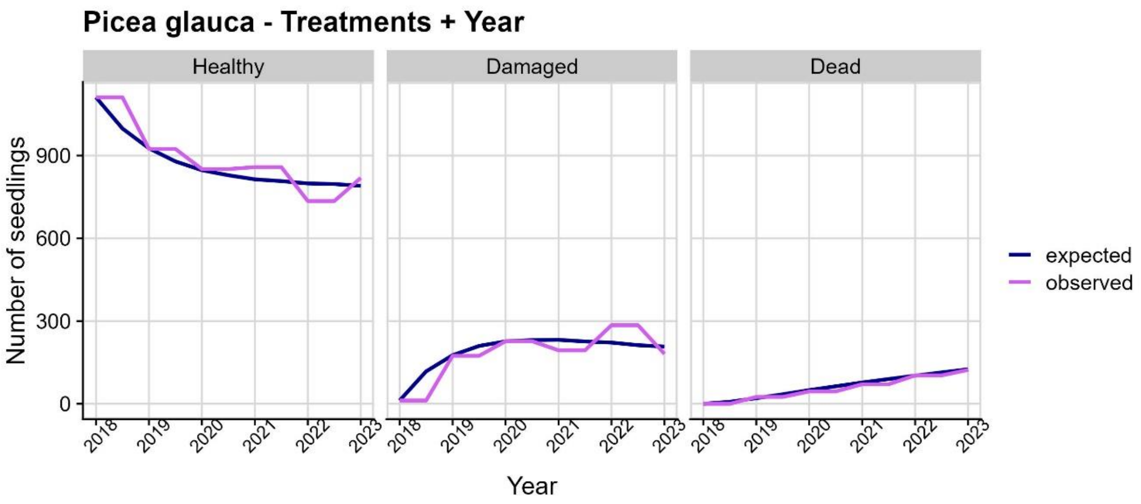

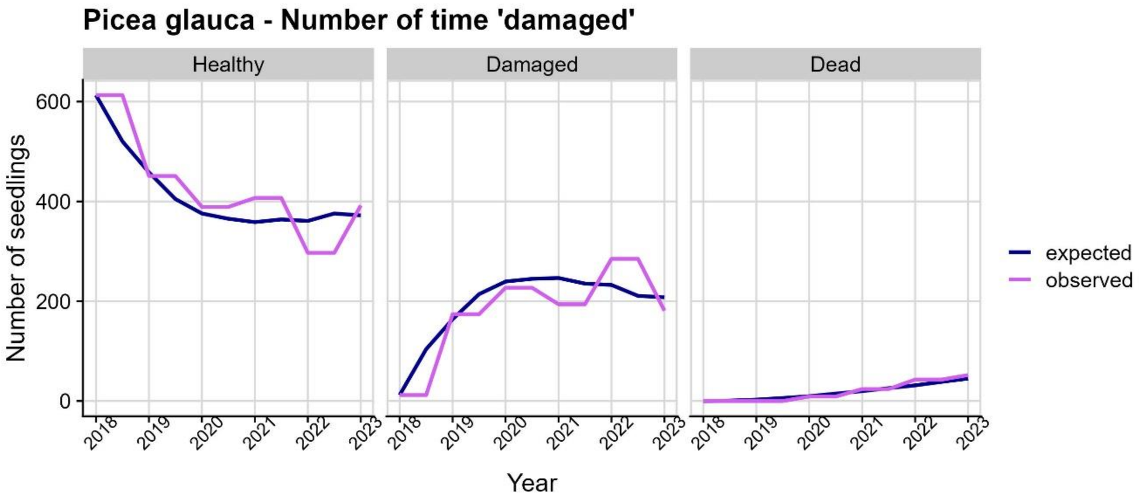

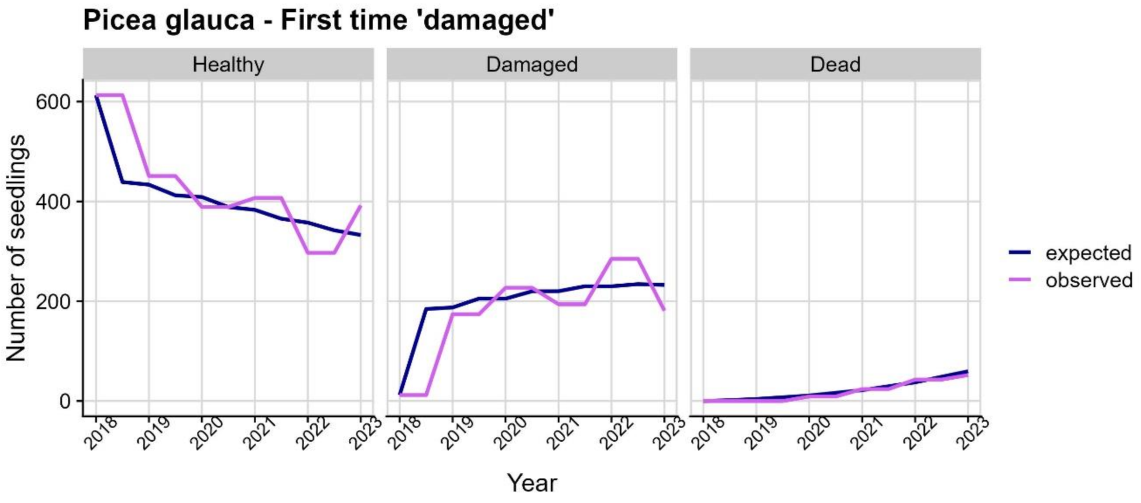

Figure S4. Graphical assessment of models fit for *Picea glauca*.

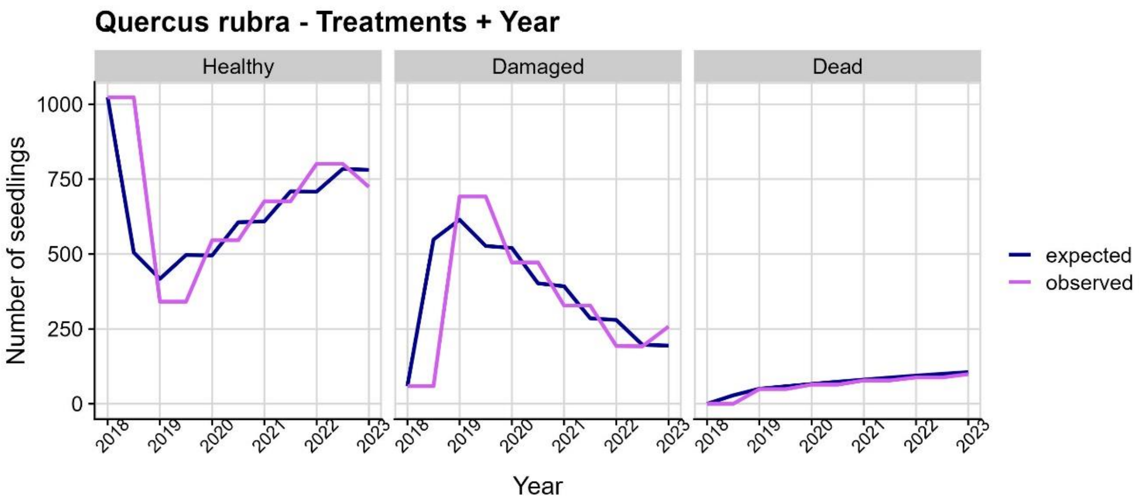

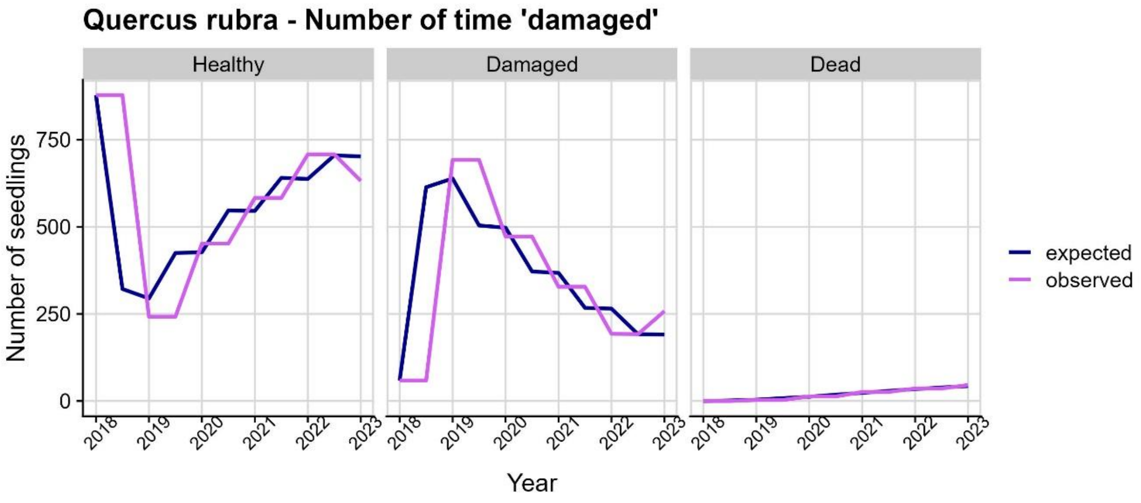

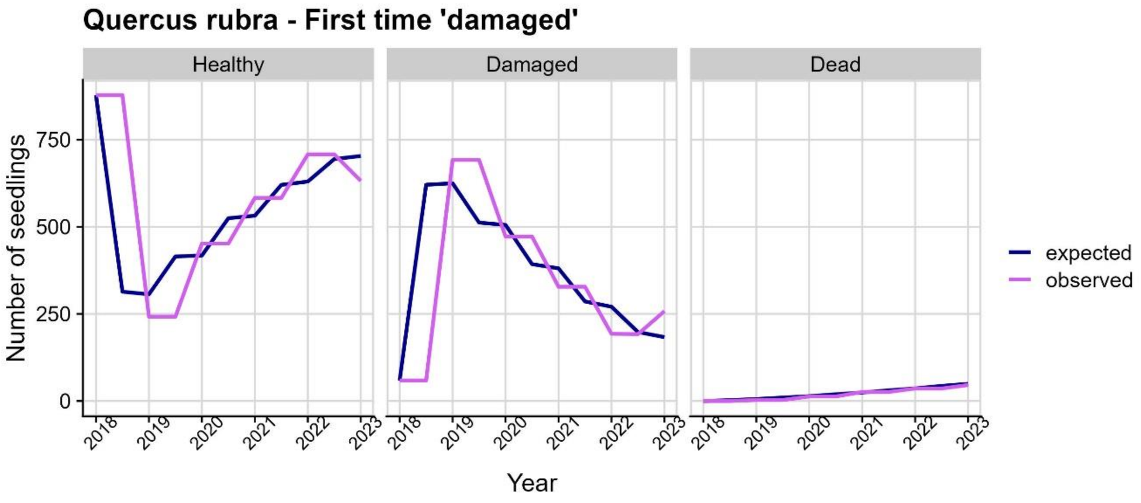

Figure S5. Graphical assessment of models fit for *Quercus rubra*.

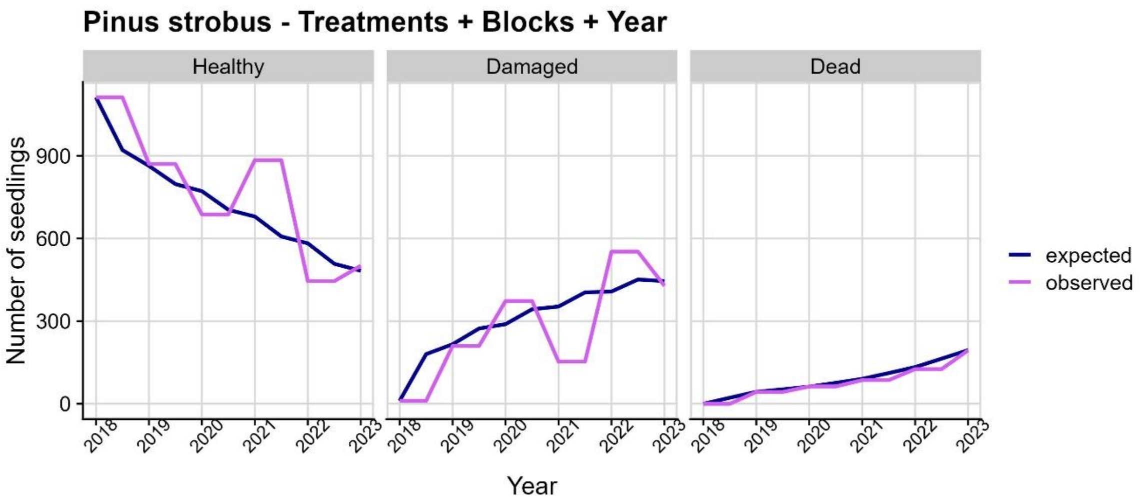

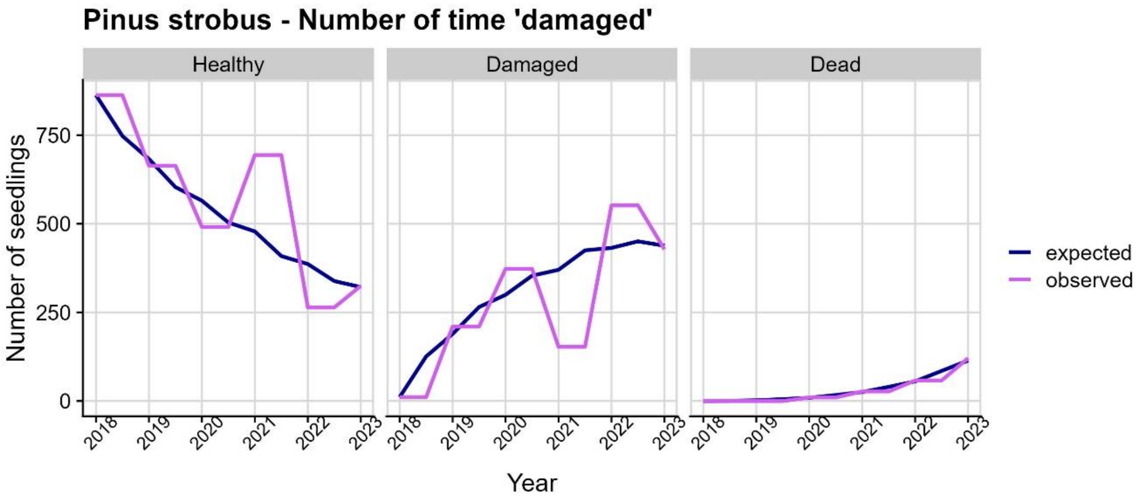

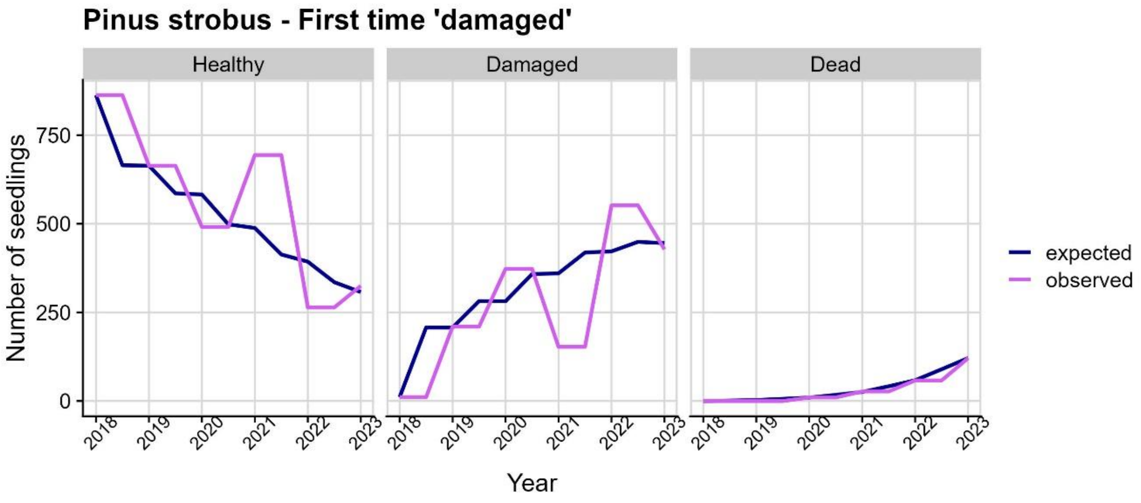

Figure S6. Graphical assessment of models fit for *Pinus strobus*.

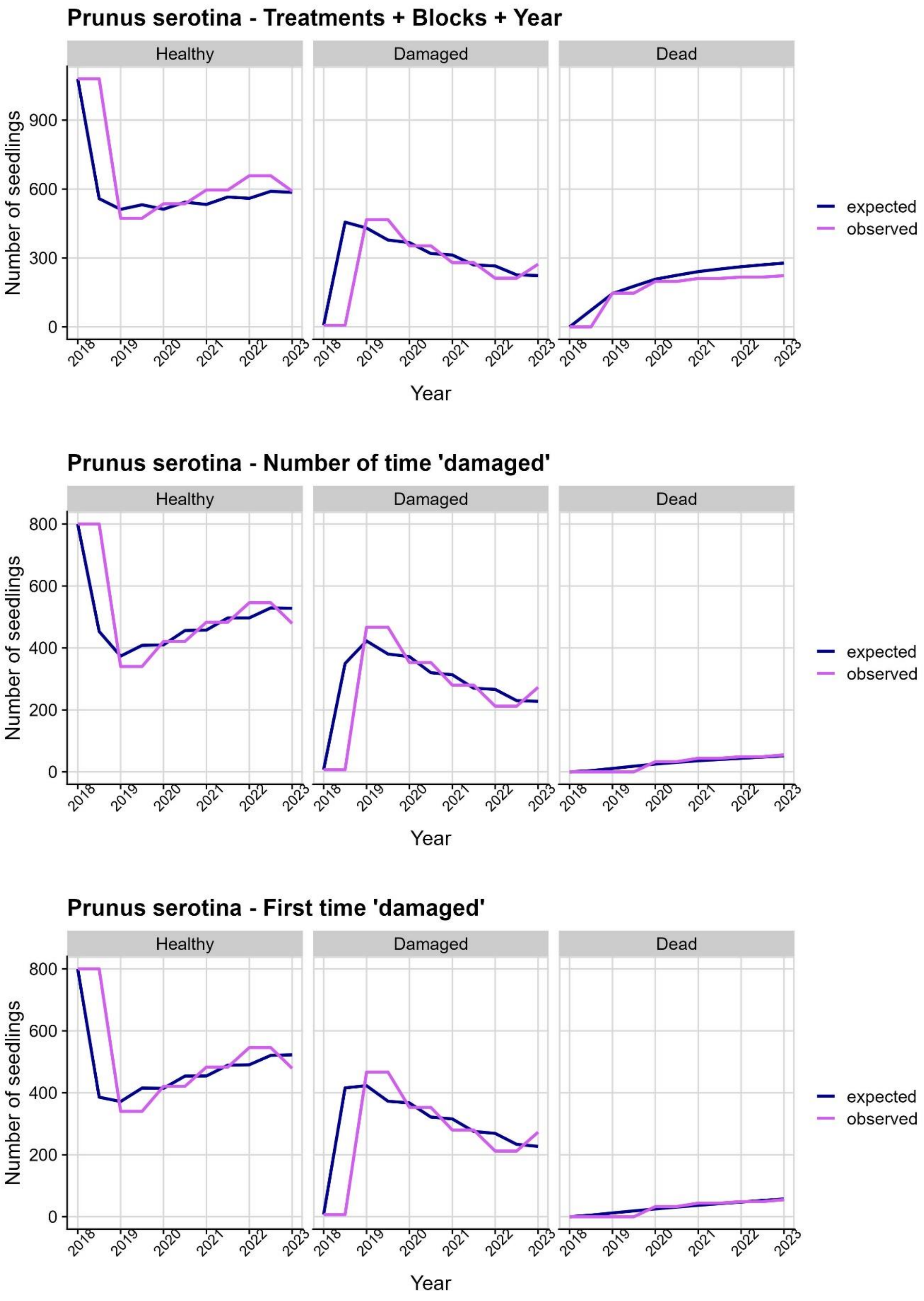

Figure S7. Graphical assessment of models fit for *Prunus serotina*.

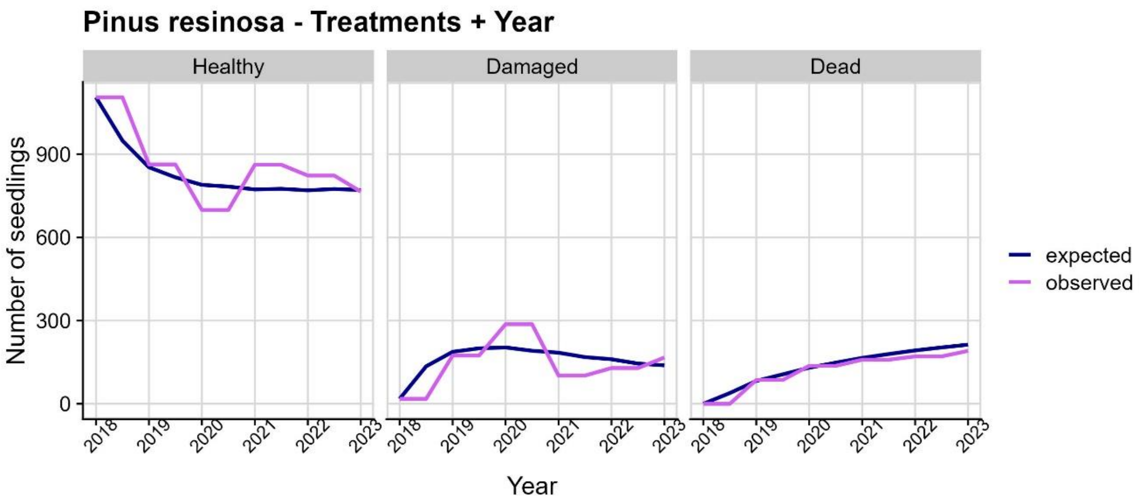

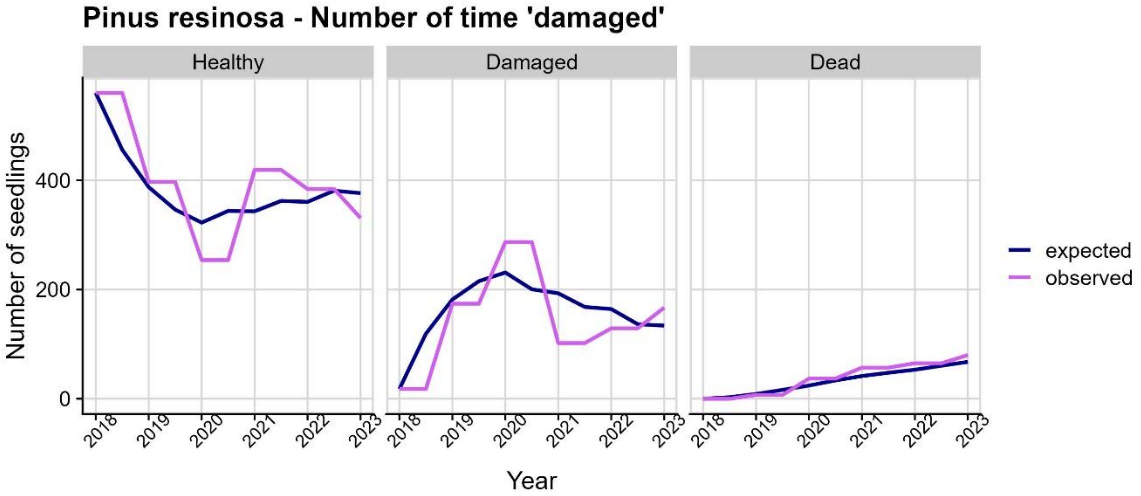

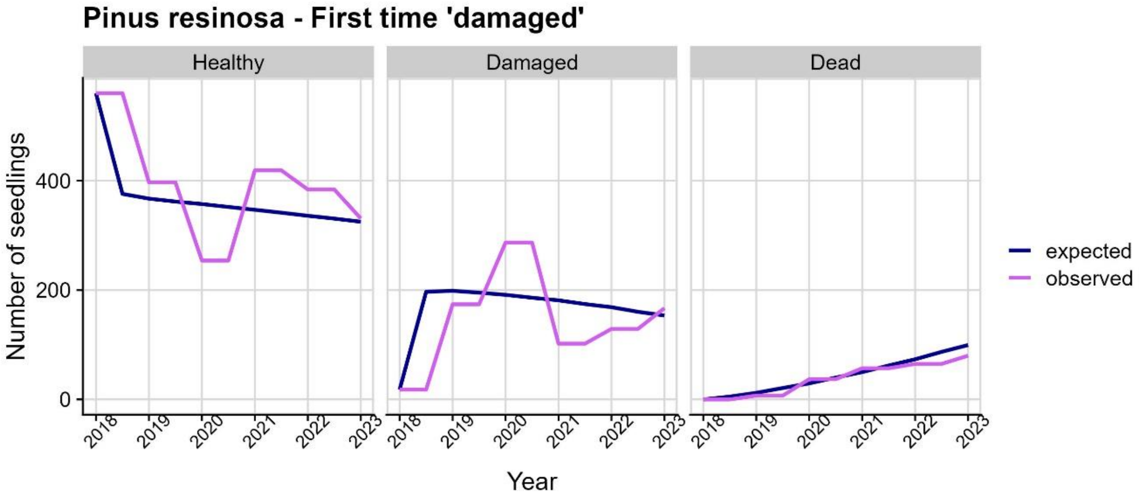

Figure S8. Graphical assessment of models fit for *Pinus resinosa*.

References

Gentleman, R. C., J. F. Lawless, J. C. Lindsey, and P. Yan. 1994. Multi-state Markov models for analysing incomplete disease history data with illustrations for HIV disease. Stat Med 13:805-821
